# Association of early life anti‐pathogen immune responses with infant food sensitisation and allergy: An elastic‐net stability‐selection approach

**DOI:** 10.1111/pai.70513

**Published:** 2026-10-22

**Authors:** Ellie McDonald, Sofie Biering‐Sørensen, Laure F. Pittet, Nigel Curtis, Nicole L. Messina

**Affiliations:** ^1^ Infectious Diseases Group Murdoch Children's Research Institute Parkville Victoria Australia; ^2^ Department of Paediatrics The University of Melbourne Parkville Victoria Australia; ^3^ Immunology, Vaccinology, Rheumatology and Infectious Diseases Unit Geneva University Hospitals and Faculty of Medicine Geneva Switzerland; ^4^ Department of Infectious Diseases The Royal Children's Hospital Melbourne Parkville Victoria Australia

**Keywords:** allergy, atopy, cytokine, innate immune, penalised regression

## Abstract

**Background:**

The development of food sensitisation (FS) and food allergy (FA) reflects complex interactions between genetic, environmental and immunological factors, which conventional univariate approaches may fail to capture. This exploratory study applied penalised regression to determine whether early‐life immune responses to common pathogens are associated with infant FS and FA.

**Methods:**

At 1 year of age, FS was determined by skin prick test and FA by allergy/tolerance history and oral food challenge. Elastic net regression (ENR) was applied to cytokine response data (13 cytokines) from whole blood stimulation (11 microbial stimuli) collected at 6 months of age (*n* = 147), together with longitudinal clinical data from MIS BAIR trial infants. Stably selected variables (0.75) were analysed by multivariable logistic regression to explore the direction of association of the stably selected variables.

**Results:**

For FS, 13 cytokine‐stimulant pairs were stably selected in cytokine‐only ENR models. After including early‐life clinical factors, four pairs were retained. Two were inversely associated with FS (*Haemophilus influenzae* IL‐1Ra and Pam3CSK4 MIP‐1α). Two were positively associated (*Streptococcus pyogenes* IL‐1Ra and peptidoglycan IFN‐γ). For FA, four cytokine‐stimulant pairs were selected in cytokine‐only ENR models. Two were positively associated with FA (*Escherichia coli*‐stimulated MCP‐1 and *S. pyogenes* IP‐10), and two inversely associated (Pam3CSK4 IL‐1Ra and *H. influenzae* IL‐1Ra). No cytokine responses were selected after inclusion of clinical factors in the FA model. The most consistently selected clinical factors were infant eczema for FS and topical corticosteroid treatment by 6 months for FA likely due to disease severity.

**Conclusion:**

Early‐life cytokine responses to pathogens associated with FS and FA at 1 year were identified through stability selection. While hypothesis‐generating, findings were consistent with a role for early immune perturbation in the development of food sensitisation.

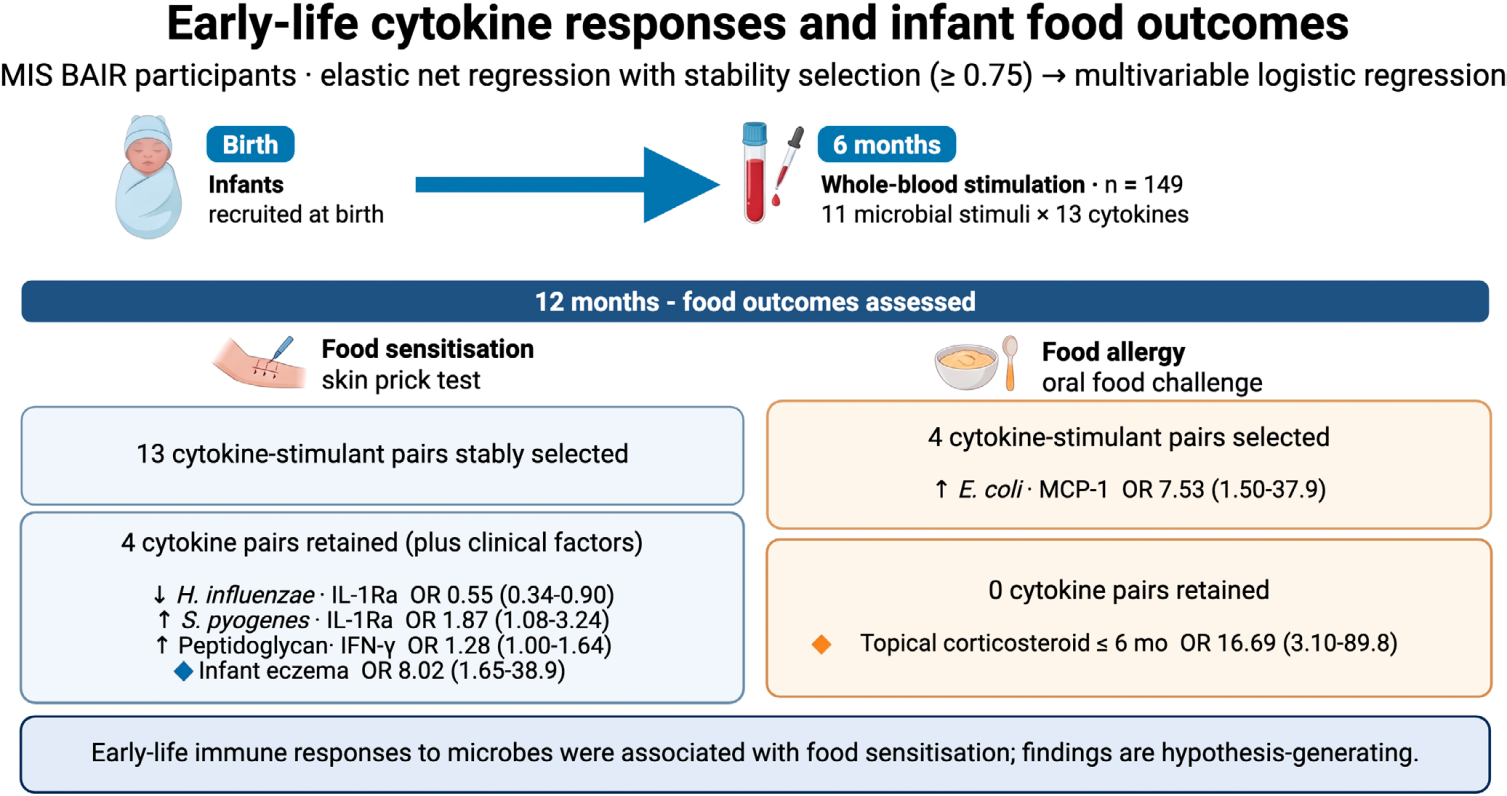

## Graphical abstract

Stability selection using penalised regression was applied to cytokine responses in microbially stimulated whole blood samples collected at 6 months, together with clinical factors, to identify associations with food sensitisation and food allergy at 1 year. Although hypothesis‐generating, the findings were consistent with early immune perturbation contributing to the development of food sensitisation. Created in BioRender. https://BioRender.com/voracad

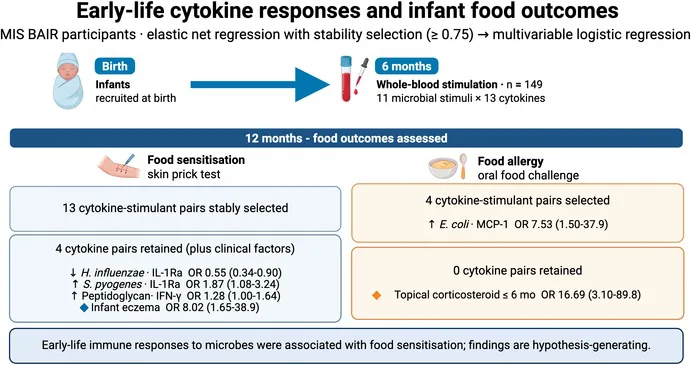

**Key message**

Penalised regression with stability selection identifies early‐life immune responses and clinical factors that are reproducibly associated with food sensitisation and allergy while accounting for correlation among candidate predictors. Infant cytokine responses to microbial stimuli showed differential associations with food sensitisation and allergy. Interleukin‐1 receptor antagonist (IL‐1Ra) responses were associated in a pathogen‐dependent manner. Infant eczema and topical corticosteroid treatment were the most consistently selected clinical factors. These hypothesis‐generating findings require confirmation.

## 1 INTRODUCTION

Food allergies (FA) are a major public health concern, with prevalence increasing globally, particularly in countries with Westernised lifestyles.^1^, ^2^, ^3^ Atopic sensitisation is a hallmark of IgE‐mediated FA; however, 30%–90% (depending on factors such as allergen, test cut‐off, age) of individuals with food allergen sensitisation (FS) do not have FA.^4^ Hypotheses proposed to explain the increasing prevalence of FA include the ‘Old Friends Hypothesis’, which posits that reduced microbial exposure during infancy impairs the establishment of immune tolerance leading to immune dysfunction and subsequent allergic disease.^5^ In addition to skewing of immune responses toward T helper (Th) 2 dominance, the presence of allergy‐associated immune signatures in unstimulated and endotoxin lipopolysaccharide (LPS)‐stimulated cells and in cord blood suggests baseline immune dysregulation in allergic individuals.^6^, ^7^

Efforts to develop preventative interventions have been hampered by an incomplete understanding of the complex interplay between genetic, environmental and immunological factors in the development of sensitisation and allergy to food antigens.^8^ Using a penalised regression with stability selection, integrating demographic, clinical, and environmental factors with early life immune responses, this exploratory study aimed to describe anti‐pathogen cytokine responses and early‐life factors reproducibly associated with IgE‐mediated FS and FA in infants, as a basis for future hypothesis‐driven study.

## 2 METHODS

### 2.1 Participants

This study included data from participants in the Melbourne Infant Study: BCG for Allergy and Infection Reduction (MIS BAIR) randomised controlled trial (RCT) (NCT01906853). The trial protocol, including inclusion criteria, data collection, FS and FA assessment have been published previously and an overview is presented in the Supporting Methods S1 section.^9^, ^10^, ^11^ Participants were included if they had consented for future analysis of data and had a valid skin prick test (SPT) at the 1 year study visit, and for (i) cytokine analyses had a whole blood cytokine response to at least *Escherichia coli* and Nil stimulation, and (ii) clinical data analyses were not missing included clinical data variables. Participants from both the intervention and control arms were included.

### 2.2 IgE‐mediated food sensitisation and allergy outcomes

An infant was classified as FS if they had a positive SPT (wheal size ≥2 mm greater than the negative (saline) control) for any core food allergen (peanut, raw egg white, cow's milk, hazelnut, shellfish, cashew nut, and sesame).^10^ Participants with FS were classified as FA if they had clinically defined or oral food challenge (OFC) proven allergy to any core food allergen. Participants were classified as non‐FS (and non‐FA) if they were SPT negative for all core food allergens and had a valid positive (histamine) control.^10^ FS participants who were not allergic to food allergens (FS only) were excluded from statistical analysis of FA outcomes.

### 2.3 *Ex vivo* cytokine responses

All MIS BAIR participants were invited to take part in the 6‐month visit and associated blood collection. A subset of participants who provided blood at the 6‐month visit had samples suitable for in vitro stimulation assay cytokine analysis (e.g. sufficient blood collected, sample collected during in vitro stimulation processing windows).^12^, ^13^ For the subset of infants who attended the 6‐month study visit, whole blood was stimulated ex vivo with a panel of microbial stimuli (7 heat‐killed pathogens, 4 Toll‐like receptor (TLR) agonists, details in Supporting Methods S1). Cytokine secretion analysed by multiplex assay was described previously (including inclusions/exclusion criteria) and is presented in the Supporting Methods S1.^12^, ^13^

### 2.4 Data analysis

Data analyses were conducted using Stata version 18.5 (StataCorp LLC, USA) and R using R Studio (2022.07.1 + 54),^14^ R (version 4.5.12025‐06‐13 ucrt), mice,^15^ glmnet,^16^ brglm2,^17^ mitools and car.^18^ Details of data processing are provided in the Supporting Methods S1 section, and an overview of the analysis processing structure is provided in Figure S1.

### 2.5 Missing data

Prior to analysis, cytokine responses were log‐transformed. Missing cytokine values were multiply imputed using chained equations (MICE), with *m* = 50 imputations and 10 iterations by predictive mean matching (5 donors). The predictor matrix was constructed with quickpred (minimum absolute correlation 0.25), forcing inclusion of the outcome. Convergence and imputed‐versus‐observed distributions were inspected graphically before proceeding. All subsequent steps were carried out within each completed dataset and pooled across imputations.^15^, ^19^ Due to high correlation across stimuli (Spearman correlation coefficient ≥0.9, data not shown), only macrophage migration inhibitory factor (MIF) and monokine induced by gamma interferon (MIG, CXCL9) responses to *E. coli* and peptidoglycan (PEPG) were included in regression models.

### 2.6 Variable selection by elastic‐net stability selection

For each outcome we fitted two model variants that differed only in the pool of variables offered to selection: a cytokine‐only model, in which only the stimulated cytokine responses were eligible for selection, and a cytokine plus clinical model. The cytokine plus clinical model additionally offered a set of a priori clinical and environmental variables spanning infant, maternal and household domains (e.g. early‐life eczema and topical corticosteroid use, delivery and feeding, maternal education, medication and pregnancy exposures, and household composition) as well as BCG status (i.e. the MIS BAIR intervention). In the cytokine‐only models, two clinical variables (infant sex and BCG status) were included as imputation predictors only and were not eligible for selection. This yielded four models in total: FS and FA, each cytokine‐only and cytokine plus clinical.

Both outcomes (FS and FA) were binary and modelled by logistic regression with a logit link. Variables were selected by stability selection with elastic‐net penalised logistic regression,^20^, ^21^ which combines L1 (lasso) and L2 (ridge) penalties. Fifty bootstrap resamples were drawn from each of the 50 multiply‐imputed datasets, giving 2500 elastic‐net fits per model. Within each fit, the mixing parameter alpha (α) was tuned over 0.1, 0.3, 0.5, 0.7, and 0.9 by 5‐fold cross‐validation maximising the area under the receiver‐operating‐characteristic curve (AUC), and the penalty λ was set to its cross‐validated‐optimal value (λ.min) (cv.glmnet, glmnet package, R). Variables with non‐zero coefficients at λ.min were recorded as selected. Continuous variables were standardised internally within each imputed, resampled dataset, and categorical variables expanded to indicator columns were mapped back to their source variable. For each variable, a selection frequency was calculated as the proportion of the 2500 fits in which it was selected. A variable was declared stable if this frequency reached a pre‐specified threshold of 0.75.

### 2.7 Final models, effect estimates and diagnostics

For each model, variables meeting the pre‐specified inclusion threshold were analysed by multivariable logistic regression on each imputed dataset refitted with Firth's penalty (brglm2) to reduce small‐sample and separation bias,^22^ and pooled using Rubin's rules (mitools). As descriptive summaries of model fit, we report each model's in‐sample area under the ROC curve and its apparent calibration slope, averaged across imputations. For each final model we report the events‐per‐variable (EPV) ratio and, where more than one variable was retained, variance inflation factors (VIF) from an ordinary logistic fit of the same variables.

### 2.8 Ethics

Ethical and governance approval for MIS BAIR was obtained from the Royal Children's Hospital Melbourne (RCH) Human Research Ethics Committee (HREC, No. 33025) and the Mercy Health HREC (No. R12‐28), with additional governance approval from Barwon Health and St John of God, Geelong, Victoria. Further ethical approval for this exploratory study was approved by RCH HREC (No. 102949).

## 3 RESULTS

Among the 1293 infants in the MIS BAIR trial (including 21 twins), 960 had a FS result, of whom 147 also had valid 6‐month cytokine response data and 813 had clinical data only (Table 1 and Table S1). Demographic and early‐life factors were similar between the participants with and without cytokine response data with some exceptions. Compared with the clinical‐only group, the cytokine subgroup had higher proportions of BCG received as part of the trial, male gender, breastfeeding for longer than 3 months and 3 or 4 household inhabitants. Conversely, compared with the clinical‐only group, the cytokine subgroup had lower proportions of vitamin D supplementation in hospital and no siblings at birth (Table 1 and Table S1). In the cytokine subgroups, the prevalence of FS and FA was 17.0% and 8.3%, compared with 18.7% and 9.5% in the clinical‐only group. Egg was the most common food allergen for both FS (52% of FS infants in the cytokine subgroup; 57% in the clinical‐only group) and FA (73% and 75%, respectively). Sensitisation to multiple food allergens occurred in 24% (6/25) of infants in the cytokine subgroup and 34% (51/151) in the clinical‐only group. Allergy to multiple foods occurred in 18% (2/11) and 26% (20/77), respectively (Table S1). FS and FA to allergens other than egg (but not excluding egg) was 10% and 3%, respectively.

**TABLE 1:** Characteristics of participants in the FS and FA combined (clinical + cytokine) models.

	Clinical data only (no cytokine)	Food sensitisation (FS)	Clinical food allergy (FA)
	*N* = 813	*N* = 147	*N* = 133
		Cytokine + clinical	Cytokine + clinical
Male	404 (49.8%)	83 (56.5%)	75 (56.4%)
Received BCG as part of trial	412 (50.8%)	89 (60.5%)	80 (60.2%)
Age (days) at 6 m blood collection, median (IQR)	—	197.0 (37.5)	197.0 (36.0)
Age (days) at 1y clinic, median (IQR)	406.0 (46.0)	408.0 (30.5)	408.0 (30.0)
Sensitised to food allergen (FS) at 1 year	152 (18.7%)	25 (17.0%)	11 (8.3%)
Clinical allergy to any food allergen (FA) at 1 year			
Yes	77 (9.5%)	11 (7.5%)	11 (8.3%)
No	661 (81.3%)	122 (83.0%)	122 (91.7%)
Missing	75 (9.2%)	14 (9.5%)	0 (0.0%)

*Note*: *N* (%) unless otherwise indicated. Light blue refers to Food Sensitisation (FS) and dark blue refers to Food Allergy (FA).

Abbreviations: IQR, interquartile range; m, month; y, year.

To investigate whether early‐life immune responses were associated with subsequent development of FS and FA by 1 year of age, we analysed the ex vivo cytokine responses at 6 months of age to a range of killed pathogens and TLR agonists. Due to shared pathogen‐associated molecular patterns (PAMPs) between microbial species and convergence of signalling pathways following TLR activation, immune responses to different pathogens and TLR agonists are often highly correlated. Elastic‐net regression is a penalised logistic regression method well suited to data with many correlated candidate variables, as it performs variable selection and coefficient shrinkage simultaneously. We therefore used ENR within a stability‐selection framework (variables with at least 75% retention across replicates were considered stable) to identify early life anti‐pathogen cytokine responses that most consistently distinguished infants who developed FS and FA by 1 year of age from those who remained non‐sensitised.^23^

In the FS cytokine model, the most frequently selected responses were lipopolysaccharide (LPS)‐stimulated interleukin (IL)‐10, PEPG‐stimulated interferon‐γ (IFN‐γ), *E. coli*‐stimulated monocyte chemoattractant protein‐1 (MCP‐1) and *Haemophilus influenzae*‐stimulated IL‐1 receptor antagonist (IL‐1Ra) (selection frequencies 0.92–0.95 (Figure 1, Table 2)). Across stimulants, the responses selected most often involved PAM3CSK4 and among cytokines most often included IL‐1Ra (Figure 1, Table 2). Despite high selection frequencies, *H. influenzae* IL‐1Ra response was of greatest interest in the pooled refit, being inversely associated with FS (OR 0.44, 95% CI 0.24–0.83); the remaining responses had wide intervals (Table 2, Figure 2). For the FA cytokine model, only four responses were stably retained: *E. coli* MCP‐1, *Streptococcus pyogenes*‐stimulated  IFN‐γ induced protein 10 (IP‐10), Pam3CSK4 IL‐1Ra and *H. influenzae* IL‐1Ra (Figure 1, Table 2). Of these, *E. coli* MCP‐1 was of note in the pooled refit and was positively associated with FA (OR 7.53, 95% CI 1.50–37.88), albeit with a wide interval (Figure 2, Table 2).

**FIGURE 1:**
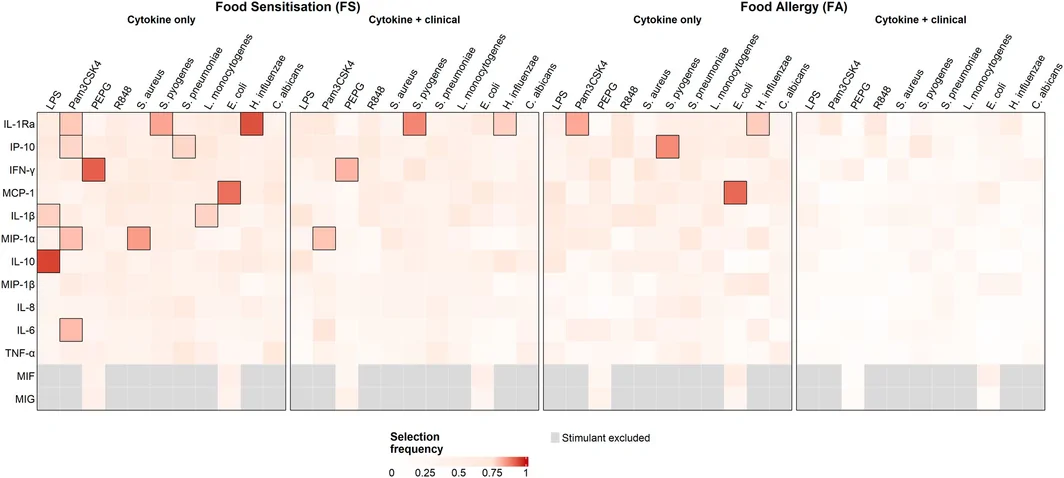
Selection frequency of anti‐pathogen cytokine responses to atopic sensitisation and food allergy. Heatmap of whole blood anti‐pathogen cytokine responses selection frequency over 4 models; atopic sensitisation, food allergy, both with and without clinical factors. MIF and MIG only had *E. coli* and PEPG responses included in ENR analysis. IFN, interferon; IL, interleukin; IL‐1Ra, IL‐1 receptor antagonist; IP‐10, IFN‐γ induced protein‐10; LPS, lipopolysaccharide; MCP‐1, monocyte chemoattractant protein‐1; MIF, macrophage migration inhibitory factor; MIG, monokine induced by IFN‐γ; MIP, macrophage inflammatory protein; Pam3CSK4, Pam3CysSerLys4; PEPG, peptidoglycan; R848, resiquimod; TNF, tumour necrosis factor.

**TABLE 2:** Stable variables, selection frequency and odds ratios.

Model	Variable	Selection frequency	OR (95% CI)	*p*
Food sensitisation (FS)				
Cytokine only	LPS: IL‐10	0.95	1.97 (0.87–4.46)	.11
	*H. influenzae*: IL‐1Ra	0.94	0.44 (0.24–0.83)	.01
	PEPG: IFN‐γ	0.92	1.18 (0.96–1.45)	.11
	*E. coli*: MCP‐1	0.90	2.66 (0.71–9.95)	.15
	*S. aureus*: MIP‐1α	0.85	2.31 (0.71–7.56)	.17
	*S. pyogenes*: IL‐1Ra	0.83	1.53 (0.98–2.41)	.06
	Pam3CSK4: IL‐6	0.80	0.54 (0.20–1.49)	.24
	Pam3CSK4: MIP‐1α	0.80	0.50 (0.19–1.28)	.15
	Pam3CSK4: IL‐1Ra	0.79	0.79 (0.53–1.18)	.25
	LPS: IL‐1ß	0.78	2.84 (0.85–9.44)	.09
	*L. monocytogenes*: IL‐1ß	0.77	1.14 (0.62–2.12)	.68
	*S. pneumoniae*: IP‐10	0.77	0.61 (0.24–1.53)	.29
	Pam3CSK4: IP‐10	0.77	0.46 (0.20–1.06)	.07
FS, Cytokine + clinical	Baby: Eczema by 6 m	0.99	8.02 (1.65–38.91)	.01
	Baby: Topical steroid treatment by 6 m	0.97	7.12 (1.40–36.17)	.02
	Mother: University level education	0.89	5.89 (1.06–32.90)	.04
	Baby: Daycare attendance	0.89	2.49 (0.78–7.93)	.12
	Mother: Antibiotics last trimester pregnancy	0.88	7.78 (1.44–41.92)	.02
	*S. pyogenes*: IL‐1Ra	0.87	1.87 (1.08–3.24)	.02
	PEPG: IFN‐γ	0.82	1.28 (1.00–1.64)	.05
	Pam3CSK4: MIP‐1α	0.79	0.62 (0.36–1.05)	.08
	Baby: Birth weight	0.78	1.00 (1.00–1.00)	.26
	*H. influenzae*: IL‐1Ra	0.78	0.55 (0.34–0.90)	.02
	Household: Cat/Dog/Livestock	0.77	1.98 (0.55–7.11)	.30
Clinical food allergy (FA)				
Cytokine only	*E. coli*: MCP‐1	0.91	7.53 (1.50–37.88)	.01
	*S. pyogenes*: IP‐10	0.87	1.58 (0.71–3.52)	.26
	Pam3CSK4: IL‐1Ra	0.83	0.77 (0.55–1.10)	.15
	*H. influenzae*: IL‐1Ra	0.78	0.67 (0.42–1.08)	.10
Cytokine + clinical	Baby: Topical steroid treatment by 6 m	0.98	16.69 (3.10–89.83)	<.01
	Baby: Eczema by 6 m	0.88	3.62 (0.69–18.94)	.13
	Baby: Daycare attendance	0.86	10.48 (1.82–60.45)	.01

**FIGURE 2:**
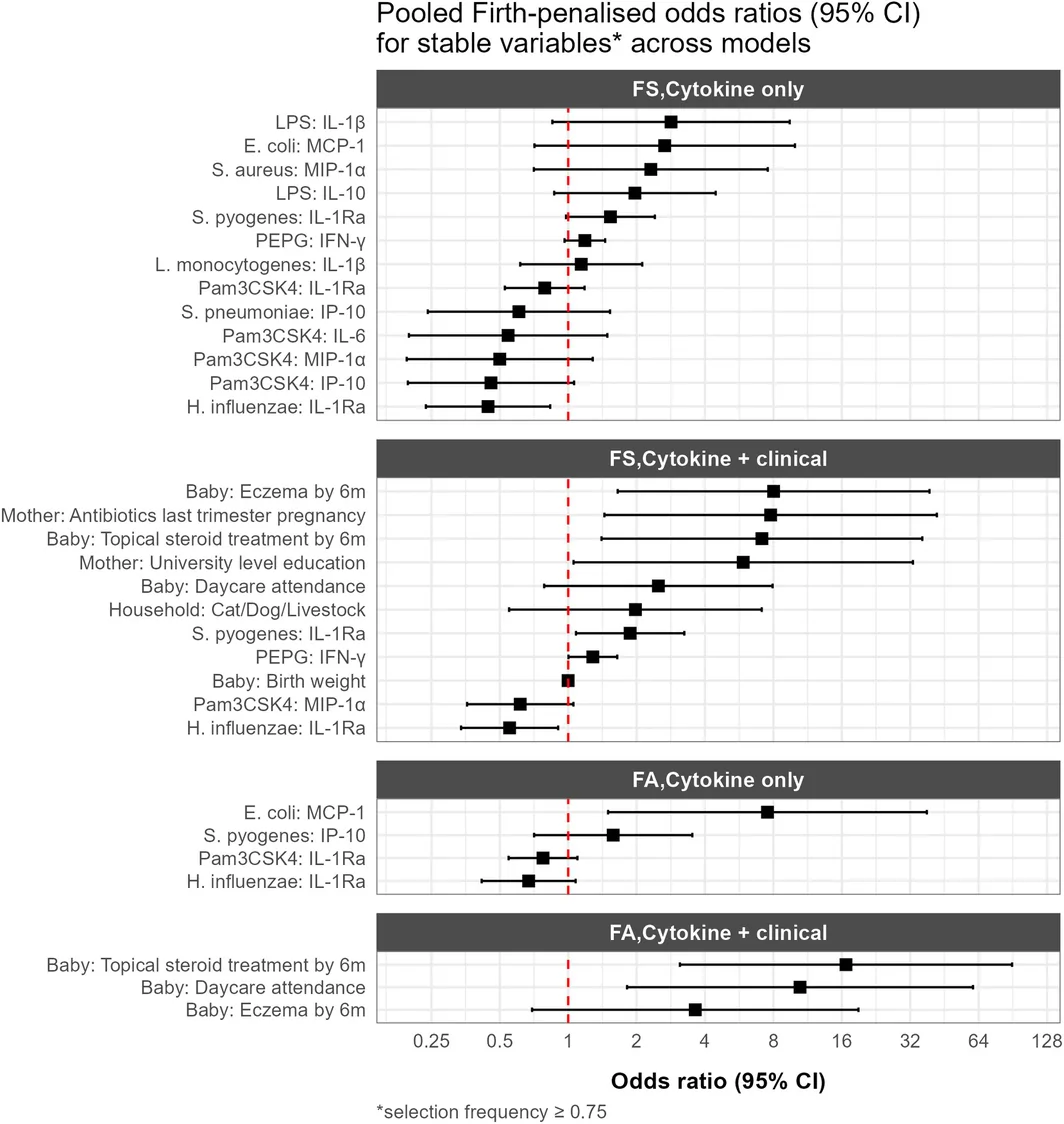
Forest plot of pooled Firth‐penalised odds ratios for stable variables across models. Forest plot of pooled Firth‐penalised odds ratios (95% confidence intervals, log scale) for variables reaching the stability‐selection threshold (selection frequency ≥0.75) in each of the four models: Food sensitisation and clinical food allergy at 1 year of age, each modelled using cytokine responses alone and using combined clinical and cytokine data. The dashed vertical line indicates an odds ratio of 1. CI, confidence interval; FA, food allergy; FS, food sensitisation; IFN, interferon; IL, interleukin; IL‐1Ra, IL‐1 receptor antagonist; IP‐10, IFN‐γ induced protein 10; LPS, lipopolysaccharide; MCP‐1, monocyte chemoattractant protein 1; MIP‐1, macrophage inflammatory protein 1; Pam3CSK4, Pam3CysSerLys4; PEPG, peptidoglycan.

Multiple demographic, early‐life exposure and other clinical risk factors have been associated with FS and FA development, many of which may influence early‐life immune response. We therefore applied ENR models combining clinical and immunological factors. Model diagnostics for cytokine only and combined cytokine and clinical models are summarised in Table 3. Because the aim was to identify stably selected factors rather than to build a prediction model, these metrics are reported descriptively and not as evidence of predictive performance. Discrimination (Apparent AUC) was moderate to high across all four models (AUC 0.85–0.93) with the strongest performance in the combined models (AUC 0.93 for FS and 0.89 for FA; Table 3). However, being calculated in‐sample and without optimism correction, this is likely to be optimistic and all models had fewer than ten events per variable (EPV 1.9–3.7). Calibration slopes exceeded one in every model (1.09–1.25), indicating that predicted probabilities were, if anything, not sufficiently extreme, indicative of the pattern expected from penalised estimation on small samples. Collinearity among retained variables was modest (maximum variance inflation factor <5 in three of four models). Across resampling, individual fits selected many variables (median 23–64 per fit) but only a small, reproducibly selected subset reached the stability threshold (3–13 variables), underscoring the instability of selection at this sample size. Elastic‐net stability selection frequencies for all variables across the four models are available in Tables S3–S6, with frequencies at or above 0.60 available in Figure S2.

**TABLE 3:** Model performance and diagnostics.

	Food sensitisation (FS)		Clinical food allergy (FA)	
	Cytokine only	Cytokine + clinical	Cytokine only	Cytokine + clinical
	*N* = 146	*N* = 146	*N* = 133	*N* = 133
Discrimination				
Apparent AUC (95% CI)	0.88 (0.79–0.94)	0.93 (0.85–0.97)	0.85 (0.67–0.94)	0.89 (0.67–0.97)
Calibration				
Calibration intercept (target 0)	−0.17	−0.13	−0.15	−0.13
Calibration slope (target 1)	1.25	1.22	1.09	1.10
Model size and selection stability				
Stable variables, *n* (*π* ≥ 0.75)	13	11	4	3
Median variables selected per run	58	64	47	23
Empty‐model runs, *n*	1	1	11	1
ENR failure rate	0.00%	0.00%	0.36%	0.36%
Sample adequacy and collinearity				
Events per variable (EPV)	1.92	2.27	2.75	3.67
Maximum VIF	5.53	1.82	1.64	1.26

*Note*: Light blue refers to Food Sensitisation (FS) and dark blue refers to Food Allergy (FA).

In the combined models, infant eczema by 6 months of age was the most frequently selected factor for FS (selection frequency 0.99), with 50% of infants with eczema by 6 months having FS at 1 year compared to 11% for children without eczema by 6 months (Table 2, Figure 3B). Infant eczema was the second most frequent for FA after topical steroid treatment by six months (0.88; Table 2, Figure 3B). FA at 1 year was over five times higher among children with either eczema by 6 months of age (27%) or topical steroid treatment at 6 months (31%) compared to those without (4% and 2%, respectively) (Figure 3B,C).

**FIGURE 3:**
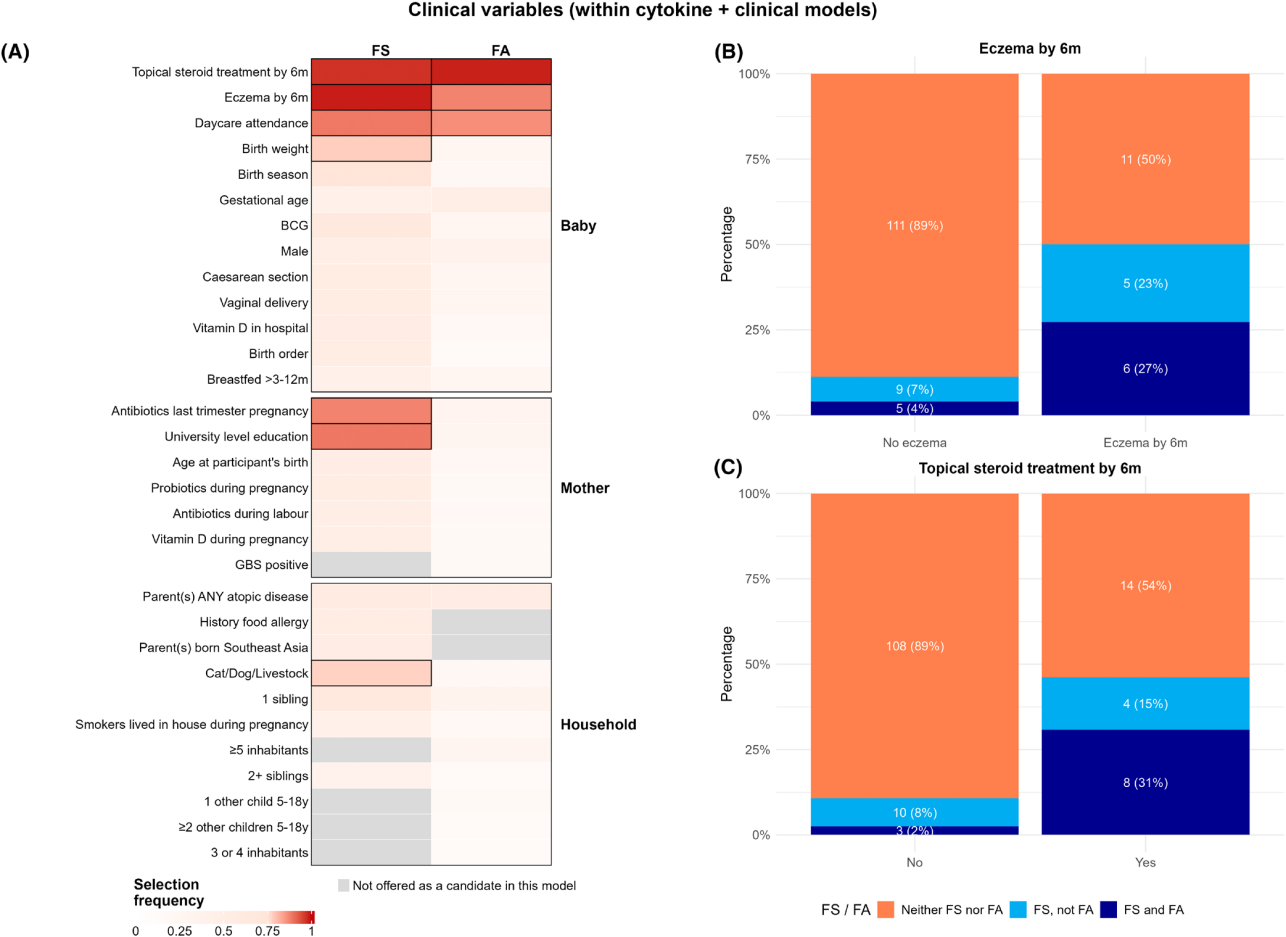
Selection frequency of early‐life factors for food sensitisation and food allergy within the clinical + cytokine models. (A) Heatmap of early life factor selection frequency. Proportion of infants with food sensitisation and/or allergy at 1 year among those (B) with and without eczema by 6 months of age; and (C) with and without topical steroid treatment by 6 months of age. BCG, Bacille Calmette‐Guérin; FS, food sensitisation; FA, Food allergy; GBS, Group B streptococcus (pregnancy screening); m, months; y, years.

Topical steroid treatment by 6 months of age, which was predominantly administered to infants with eczema, also had a high selection frequency and a positive association with both outcomes (FS OR 7.12, 95% CI 1.40–36.17; FA OR 16.69, 95% CI 3.10–89.83; Table 2). Eczema severity (determined by Patient Oriented Eczema Measure (POEM) score)^24^, ^25^, ^26^ could not be included in the ENR model due to >6% missing data but it is likely that infants with more severe eczema were more likely to receive topical steroid treatment. To determine this, we assessed the association between topical steroid treatment by 6 months of age with eczema severity in all infants with eczema (including those without cytokine data). As expected, odds ratios for steroid use increased with increasing eczema severity (Table S2).

For the FS cytokine and clinical model, although least frequently selected among the stable cytokine responses (retained 78% of models), *H. influenzae* IL‐1Ra responses had an inverse association with FS (OR 0.55, 95% CI 0.34–0.90) as it had in the cytokine‐only model (Figures 1 and 2, Table 2). Among the other stably selected cytokine responses in the combined model, *S. pyogenes* IL‐1Ra and PEPG IFN‐γ responses had the highest selection frequency and were positively associated with FS (OR 1.87, 95% CI 1.08–3.24 and OR 1.28, 95% CI 1.00–1.64, respectively) (Figures 1 and 2, Table 2). For both of these responses, the association with FS appeared to be influenced by infant eczema by 6 months (Figure 4), resulting in a stronger association in the combined model than the cytokine‐only model. For FA, the combined model retained only topical steroid treatment, eczema, and daycare attendance as stable factors (Figure 3, Table 2). Multiple cytokine responses selected in the cytokine‐only models were not retained as stable in the combined models (Figure 1, Figure 2). This is likely due to interactions between demographic and clinical factors with cytokine responses. For example, differences in *E. coli* MCP‐1 responses between infants with FS and FA at 1 year and those without appeared to be driven by different responses among infants with and without eczema by 6 months (Figure 4). For several responses, such as *S. pyogenes* IL‐1Ra and Pam3CSK4 IL‐1Ra responses, there may also be differential expression between infants who were FS without FA compared to those with FA; however, due to insufficient numbers of participants with FS and no FA, statistical modelling comparing these outcomes was not possible (Figure 4).

**FIGURE 4:**
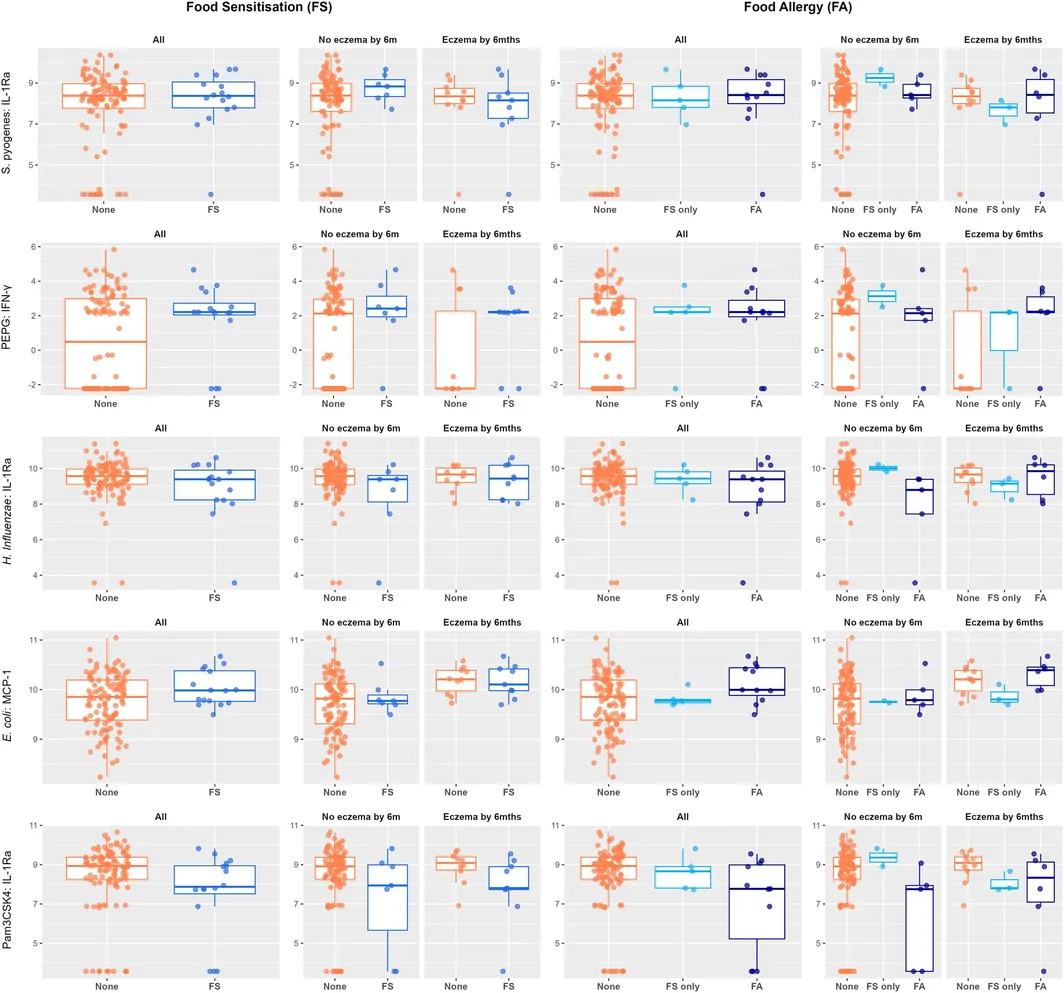
Box and scatterplots of the cytokine responses (log pg/mL) associated with food sensitisation (FS) and/or food allergy (FA) presented overall (All) or separated by infant eczema status. IFN, interferon; IL‐1Ra, interleukin‐1 receptor antagonist; IP‐10, IFN‐γ induced protein‐10; MCP‐1, monocyte chemoattractant protein‐1; Pam3CSK4, Pam3CysSerLys4; PEPG, peptidoglycan.

## 4 DISCUSSION

In this exploratory study, we used elastic‐net penalised logistic regression with stability selection to identify antimicrobial cytokine responses associated with FS and clinical FA at 1 year of age, in the context of early‐life exposure clinical factors.

Allergy is an immune‐mediated disease which, in addition to IgE sensitisation and Th2 skewing of the immune response, has been associated with a hyperinflammatory phenotype.^6^, ^27^, ^28^ Our study included multiple proinflammatory cytokines (e.g. IL‐6, TNF‐α), however stably selected cytokines were largely chemokines (MCP‐1, MIP‐1α, IP‐10), the anti‐inflammatory IL‐1Ra, with lesser representation of IL‐1β. The role of the IL‐1 pathway in allergy appears context dependent. IL‐1β responses have been shown to be higher in allergic and atopic donors, with suppression of these responses, including through IL‐1Ra, being protective against IgE mediated food allergy.^29^, ^30^, ^31^, ^32^ However, in a recent murine model, while reducing allergen specific IgE responses, IL‐1Ra or blockage of the IL‐1 pathway (through Anakinra treatment) may promote anaphylactic responses.^33^ It is unclear why differential associations were found with IL‐1Ra induced by *S. pyogenes* and *H. influenzae* responses, however it may be related to differing interactions with clinical risk factors (e.g. eczema) and allergic immune pathways. For the *Streptococcus* genus more broadly, skin and gut microbiome studies have shown increased *Streptococcus* abundance in the gut and skin of infants with FA and eczema, with some studies showing dysbiosis as early as 3 days of age.^34^, ^35^, ^36^, ^37^ In contrast, several studies have shown reduced abundance of *Streptococcus* in the oral microbiome of food allergic children.^38^ *Streptococcus* species, such as *S. thermophilus* are also included in probiotic and prebiotic infant formulas, however, the impact of their inclusion on infant development of allergic diseases is unclear.^39^, ^40^ For *H. influenzae*, there is limited data on the association between this pathogen and allergic diseases. In a murine allergic airway disease model, *H. influenzae* reduced Th2 type cytokine eosinophilic inflammation, however it also increased neutrophil influx and IL‐17 responses and effects on the IL‐1 pathway were not assessed.^41^

TLRs have diverse roles in the regulation of the immune system, including promotion of epithelial barrier function and the maturation, function and regulation of immune cells. As a Gram‐negative bacterium, *H. influenzae* is a strong inducer of TLR4 activation. Consistent with FS being associated with reduced anti‐inflammatory *H. influenzae* responses, previous studies have also shown higher pro‐inflammatory cytokines and IL‐10 responses to the TLR4 agonist, LPS, among egg‐allergic infants and in cord blood of infants who developed FA by 1 year of age.^6^, ^7^, ^28^, ^42^ This TLR‐4 induced proinflammatory phenotype is also supported by the positive association of proinflammatory *E. coli* and LPS responses with FS and FA in our model.

In addition to *S. pyogenes*, a potent inducer of TLR2 activation, our study found consistent selection of cytokine responses to TLR2 agonists. Notably, while *S. pyogenes* and PEPG were positively associated with FS (and *S. pyogenes* IP‐10 with FA), responses to the TLR1/2 agonist Pam3CSK4 were negatively associated. These divergent associations may reflect the differential activation pathways induced by these TLR2 agonists, as PEPG is sensed through TLR2 and nucleotide‐binding oligomerisation domain (NOD) receptors, whereas Pam3CSK4 is a TLR1/2 agonist.^43^ A role for TLR2 in allergic diseases has been supported by animal models and cohort studies; however, whether activation of this TLR promotes food antigen tolerance or allergy is yet to be determined.^44^ Consistent with the negative associations of the TLR1/2 agonist Pam3CSK4 with FS and FA across the ENR models in our study, a study of children with nut and/or egg allergy found reduced *TLR2* and *TLR4* whole blood gene expression compared to non‐allergic infants.^45^ In contrast, one study found increased pro‐inflammatory cytokine responses to Pam3CSK4 (as well as LPS) in cord blood of infants who developed FA by one year^42^ and in a murine model, induction of tolerance was TLR2 independent and inhibited by low‐dose oral Pam3CSK4 treatment.^46^, ^47^, ^48^ Overall, there is a likely interplay between TLR responses and infant FS and FA, but mechanisms and implications of this require further research.

Early‐life exposures and risk factors can influence measured immune responses and may also reflect underlying immune dysregulation that contributes to the development of allergic disease. Cohort studies have consistently found positive associations between eczema and FS and/or FA, with eczema often preceding FA/FS, supporting a causal role or common pathway in what is termed the ‘atopic march’.^49^, ^50^, ^51^ Consistent with this, eczema by 6 months was a major feature positively associated with FS and FA in the cytokine and clinical combined models in our study. In our study, topical steroid use by 6 months of age was associated with increased odds of both FS and FA by 1 year. However, infants who received topical steroid treatment by 6 months of age were also more likely to have had more severe eczema than those who did not. Therefore, the stable selection of early‐life steroid treatment in our study was likely confounded by eczema severity among those who received early‐life topical steroid treatment rather than a causal relationship between the topical steroid treatment and increasing FS and FA risk.

The strengths of our study include the use of penalised regression with stability selection, which handles multicollinearity, performs variable selection and coefficient shrinkage simultaneously, and can accommodate many candidate predictors relative to the number of observations.^16^ Further strengths include objective outcome measures of FS and FA, the use of detailed prospectively collected data from birth and measurement of responses to a range of common early‐life pathogens and TLR agonists, and both pro‐ and anti‐inflammatory cytokines in early life.

Our study has several limitations. Most importantly, the relatively low number of outcome events meant all models had fewer than 10 events per variable; selection is therefore unstable and the reported effect estimates, being apparent (in‐sample) and fitted on the same data used for selection are optimistic, without opportunity for optimism‐correction or external validation. Penalisation does not overcome the limited sample size; accordingly, all associations are hypothesis‐generating and require confirmation in independent, adequately powered cohorts. The low participant numbers also precluded sub‐group analysis by eczema status at 6 months and by food allergen. The low proportion of participants with FS without FA, due in part to the MIS BAIR study design which limited the number of food challenges per infant, prevented modelling for features to differentiate FS from FA. Outcomes were IgE‐mediated (skin prick test positive, with FA confirmed by oral food challenge) and measured at 1 year. The findings therefore do not extend to non‐IgE‐mediated allergy, and 1‐year outcomes, predominantly egg allergy driven, may be transient. Persistent and allergen‐specific outcomes (e.g. peanut) could not be distinguished. Finally, participants from both trial arms were included, therefore adjustment by trial arm was not done. This was partially addressed via BCG randomisation status being included as a factor in the multiple imputation process, as well as a candidate variable in the stability selection processing. In addition, prior MIS BAIR analyses did not find any significant effect of BCG vaccination on the allergic outcomes studied here, making substantial confounding by trial arm unlikely.^10^

Our exploratory study showed that a number of early‐life anti‐pathogen cytokine responses are associated with FS and FA at 1 year. These response profiles in early life support the concept of immune dysregulation (including the innate immune system) contributing to the atopic march. Combining clinical and cytokine data yielded additional stable signals compared with cytokine‐only models, supporting the value of integrated approaches for generating hypotheses about early‐life determinants of FA.

## AUTHOR CONTRIBUTIONS

**Ellie McDonald:** Formal analysis; visualization; writing – original draft; writing – review and editing; methodology; data curation; software; conceptualization; validation. **Sofie Biering‐Sørensen:** Writing – review and editing; formal analysis; methodology; conceptualization; data curation; software; validation. **Laure F. Pittet:** Writing – review and editing; investigation; supervision. **Nigel Curtis:** Conceptualization; methodology; investigation; supervision; writing – review and editing; writing – original draft; funding acquisition. **Nicole L. Messina:** Conceptualization; methodology; investigation; formal analysis; validation; supervision; project administration; writing – original draft; writing – review and editing; visualization; funding acquisition.

## FUNDING INFORMATION

MIS BAIR was funded by the National Health and Medical Research Council (NHMRC) of Australia (GNT 1051228; 1099676), The University of Melbourne, RCH Foundation, and the Murdoch Children's Research Institute. LFP is supported by the Swiss National Science Foundation Early Postdoc Mobility grant (P2GEP3_178155) and Ambizione grant (PZ00P3‐209050). NC is supported by a National Health and Medical Research Council (NHMRC) Investigator Grant (GNT2042979).

## CONFLICT OF INTEREST STATEMENT

The authors declare no conflicts of interest.

## Supporting information


**Figure S1.** Overview of data processing pipelines. The diagram shows the workflow for identifying early‐life factors associated with food sensitisation (FS) and clinical food allergy (FA) at 1 year of age. Two candidate‐variable sets, cytokine‐only and cytokine plus clinical, are each carried left to right through five stages: model definition, data preparation, multiple imputation, elastic‐net stability selection and effect estimation and evaluation. The two model types share a common imputation, selection and estimation pipeline, differing only in the variables offered as candidates.
**Figure S2.** Elastic‐net stability selection frequencies for all variables reaching ≥0.60 in each of the four models. Dotted lines mark the 0.60/0.70/0.75/0.80/0.90 cutoffs; the dashed line marks the pre‐specified stability threshold of 0.75. Colour and point shape both indicate the stability band.
**Table S1.** Characteristics of participants in the FS and FA combined (clinical + cytokine) models.
**Table S2.** Tabulated odds of topical steroid treatment and eczema severity by 6 months.
**Table S3.** Elastic‐net stability selection frequencies, FS—cytokines + a priori clinical.*
**Table S4.** Elastic‐net stability selection frequencies, FS—cytokines + full clinical.*
**Table S5.** Elastic‐net stability selection frequencies, FA—cytokines + a priori clinical.*
**Table S6.** Elastic‐net stability selection frequencies, FA—cytokines + full clinical.

## Data Availability

The data that support the findings of this study are available on request from the corresponding author. The data are not publicly available due to privacy or ethical restrictions.
