# Supplementary material for: Association of early life anti‐pathogen immune responses with infant food sensitisation and allergy: An elastic‐net stability‐selection approach
Source: Pediatr Allergy Immunol. 2026 Oct 22;37(10):e70513. doi: 10.1111/pai.70513 (PMC13688703; doi:10.1111/pai.70513)
Supplement: Supplementary file 1 [file PAI-37-e70513-s001.docx]

**Supplementary Methods 1.**

**Clinical data collection**

Baseline data on demographics, pregnancy, exposures and the perinatal period were collected at inclusion. At 3, 6, 9 and 12 months post randomisation, parents completed surveys which included questions about exposures, allergic reactions and doctor diagnosis of allergies, as well as questions relating to eczema (UK Williams Eczema diagnosis and Patient Oriented Eczema Measure (POEM) scores^1-3^).

**Food sensitisation and allergy testing**

Details of SPT and food allergy testing at one year of age in MIS BAIR have been described previously^4^. Briefly, infants attended a study visit during which an allergy nurse applied allergens using a QUINTIP device (Hollister-Stier Laboratories, WA), and wheal size (mean diameter) was measured at 15 min for SPT. An infant was considered sensitised for an allergen if the wheal size was ≥2 mm greater than the negative (saline) control (defined as a positive SPT). The core food allergens were peanut, raw egg white, cow's milk, hazelnut, shellfish (all from ALK, Spain), cashew nut (Greer Laboratories, Stallergenes) and sesame (ALK, USA). Sensitised infants with an uncertain allergy/tolerance history were invited to attend OFCs which were done at the RCH Allergy Clinic in accordance with to the Australasian Society of Clinical Immunology and Allergy guidelines.

***Ex vivo* cytokine responses**

Whole blood diluted 1:1 in RPMI 1640 medium (GlutaMAX Supplement, HEPES, Gibco, Life Technologies) was incubated for 20 (± 2 hours) with a range microbial stimuli. These were the heat-killed pathogens: *Escherichia coli* 1.0 × 10^6^ CFU/mL, *Haemophilus influenzae* (type B) 1.0 × 10^6^ CFU/mL, *Staphylococcus aureus* 1.0 × 10^7^ CFU/ mL, *Streptococcus pyogenes* (Group A Streptococcus, GAS) 1.0 × 10^7^ CFU/mL, *Streptococcus pneumoniae* (serotype 15C) 1.0 × 10^7^ CFU/ mL, *Listeria monocytogenes* 1.0 × 10^7^ CFU/mL, *Candida albicans* 1.0 × 10^6^ CFU/mL; and Toll-like receptor (TLR) agonists (all from InvivoGen): lipopolysaccharide (LPS) 100 ng/mL, resiquimod (R848) 3.5 μg/mL, peptidoglycan (PEPG) 10 μg/mL, and 1 μg/mL (S)-(2,3-bis(palmitoyloxy)-(2-RS)-propyl)-N-palmitoyl-(R)- Cys-(S)-Ser-(S)-Lys4OH,trihydrochloride (Pam3CSK4) or RPMI only (nil) control^5^. Secreted cytokines in supernatants were quantified on an xMAP Luminex 200 Analyser using a Bio-Plex Pro Hu Screening Panel measuring interferon-γ (IFN-γ), interleukin-1β (IL-1β), IL-1Ra, IL-6, IL-8, IL-10, IFN-γ induced protein (IP-10), monocyte chemoattractant protein-1 (MCP-1), macrophage migration inhibitory factor (MIF), monokine induced by IFN-γ (MIG), macrophage inflammatory protein-1α (MIP-1α), MIP-1β and tumour necrosis factor-α (TNF-α)^6^. When insufficient blood was available for all stimulations, a predetermined priority order was used.

**Data processing**

Clinical data variables with >5% missing data were removed from the cytokine plus clinical model. In addition, categorical clinical variables were required to have at least 5% of outcome events in every level to be offered as a selection candidate; sparser variables were dropped to avoid unstable or separated estimates. Case-wise deletion was used where variables had ≤5% missing data.

For cytokine data, values below the lower limit of extrapolation were assigned a value of half the lowest value and those above the upper limit were assigned a value of 1.5x the highest value. Cytokine data was then log transformed. Missing data (i.e., due to insufficient blood volume for all stimulations to be done) were handled using multiple imputation by chained equations (MICE) with predictive mean matching (PMM) of the log-transformed cytokine data. PMM was chosen to ensure that imputed values remained within the range of observed data. The imputation model included the unstimulated cytokine response and all relevant predictors in each model and outcome variables to meet the missing-at-random (MAR) assumption. A total of 50 imputed datasets were generated. Raw MIF and MIG responses were highly correlated across stimuli (Spearman correlation coefficient ≥0.9, data not shown). Therefore, after multiple imputation, only *E. coli* and PEPG responses for each of these cytokines was included in the ENR stability selection processing. In addition, nil (RPMI only) responses were excluded from the ENR stability selection processing.

Supplementary Figure 1. Overview of data processing pipelines.

The diagram shows the workflow for identifying early-life factors associated with food sensitisation (FS) and clinical food allergy (FA) at one year of age. Two candidate-variable sets, cytokine-only and cytokine plus clinical, are each carried left to right through five stages: model definition, data preparation, multiple imputation, elastic-net stability selection, and effect estimation and evaluation. The two model types share a common imputation, selection and estimation pipeline, differing only in the variables offered as candidates.

Supplementary Figure 2. Elastic-net stability selection frequencies for all variables reaching ≥ 0.60 in each of the four models. Dotted lines mark the 0.60 / 0.70 / 0.75 / 0.80 / 0.90 cutoffs; the dashed line marks the pre-specified stability threshold of 0.75. Colour and point shape both indicate the stability band.

**Supplementary Table 1. Characteristics of participants in the FS and FA combined (clinical + cytokine) models**

|  | **Clinical data only (no cytokine)** | **Food sensitisation (FS)** | **Clinical food allergy (FA)** |
| --- | --- | --- | --- |
|  | N=813 | N=147 | N=133 |
|  |  | Cytokine + clinical | Cytokine + clinical |
| Male | 406 (49.9%) | 83 (56.5%) | 75 (56.4%) |
| Received BCG as part of trial | 414 (50.9%) | 89 (60.5%) | 80 (60.2%) |
| Age (days) at 6m blood collection, median (IQR) | — | 197.0 (37.5) | 197.0 (36.0) |
| Age (days) at 1y clinic, median (IQR) | 406.0 (46.0) | 408.0 (30.5) | 408.0 (30.0) |
| **Sensitised to food allergen (FS) at 1y** |  | | |
| No | 661 (81.3%) | 122 (83.0%) | 122 (91.7%) |
| Yes | 152 (18.7%) | 25 (17.0%) | 11 (8.3%) |
| **Clinical allergy to any food allergen (FA) at 1y** |  | | |
| No | 661 (81.3%) | 122 (83.0%) | 122 (91.7%) |
| Yes | 77 (9.5%) | 11 (7.5%) | 11 (8.3%) |
| Missing | 75 (9.2%) | 14 (9.5%) | 0 (0.0%) |
| **Sensitised to peanut (SPT ≥2mm)** |  | | |
| No | 765 (94.1%) | 141 (95.9%) | 131 (98.5%) |
| Yes | 48 (5.9%) | 6 (4.1%) | 2 (1.5%) |
| **Sensitised to egg (SPT ≥2mm)** |  | | |
| No | 726 (89.3%) | 134 (91.2%) | 125 (94.0%) |
| Yes | 86 (10.6%) | 13 (8.8%) | 8 (6.0%) |
| Missing | 1 (0.1%) | 0 (0.0%) | 0 (0.0%) |
| **Sensitised to other food allergen (SPT ≥2mm)** |  | | |
| No | 733 (90.2%) | 134 (91.2%) | 128 (96.2%) |
| Yes | 80 (9.8%) | 13 (8.8%) | 5 (3.8%) |
| **Sensitised to multiple food allergens (SPT ≥2mm)** |  | | |
| No food sensitisation ≥2mm | 661 (81.3%) | 122 (83.0%) | 122 (91.7%) |
| 1 food only | 101 (12.4%) | 19 (12.9%) | 8 (6.0%) |
| ≥2 foods | 51 (6.3%) | 6 (4.1%) | 3 (2.3%) |
| **Sensitised to food allergens other than egg (+/- egg) (SPT ≥2mm)** |  | | |
| No | 704 (86.6%) | 132 (89.8%) | 128 (96.2%) |
| Yes | 109 (13.4%) | 15 (10.2%) | 5 (3.8%) |
| **Allergic to peanut** |  | | |
| No | 786 (96.7%) | 144 (98.0%) | 132 (99.2%) |
| Yes | 23 (2.8%) | 1 (0.7%) | 1 (0.8%) |
| Missing | 4 (0.5%) | 2 (1.4%) | 0 (0.0%) |
| **Allergic to egg** |  | | |
| No | 735 (90.4%) | 136 (92.5%) | 125 (94.0%) |
| Yes | 58 (7.1%) | 8 (5.4%) | 8 (6.0%) |
| Missing | 20 (2.5%) | 3 (2.0%) | 0 (0.0%) |
| **Allergic to other food(s)** |  | | |
| No | 793 (97.5%) | 143 (97.3%) | 129 (97.0%) |
| Yes | 20 (2.5%) | 4 (2.7%) | 4 (3.0%) |
| **Allergic to multiple foods** |  | | |
| No food allergy | 736 (90.5%) | 136 (92.5%) | 122 (91.7%) |
| 1 food only | 57 (7.0%) | 9 (6.1%) | 9 (6.8%) |
| ≥2 foods | 20 (2.5%) | 2 (1.4%) | 2 (1.5%) |
| **Allergic to food other than egg (+/- egg) (SPT ≥2mm)** |  | | |
| No | 776 (95.4%) | 143 (97.3%) | 129 (97.0%) |
| Yes | 37 (4.6%) | 4 (2.7%) | 4 (3.0%) |
| **Eczema diagnosis by 6m, UK Williams diagnostic criteria** |  | | |
| No | 689 (84.7%) | 125 (85.0%) | 116 (87.2%) |
| Yes | 124 (15.3%) | 22 (15.0%) | 17 (12.8%) |
| **Topical steroid treatment by 6m** |  | | |
| No/Unknown | 665 (81.8%) | 121 (82.3%) | 111 (83.5%) |
| Yes | 148 (18.2%) | 26 (17.7%) | 22 (16.5%) |
| **Maternal education** |  | | |
| ≤ Yr 12 | 235 (28.9%) | 36 (24.5%) | 35 (26.3%) |
| University | 578 (71.1%) | 111 (75.5%) | 98 (73.7%) |
| **At least one parent born in Southeast Asia** |  | | |
| No | 746 (91.8%) | 138 (93.9%) | 126 (94.7%) |
| Yes | 67 (8.2%) | 9 (6.1%) | 7 (5.3%) |
| **Family history of food allergy** |  | | |
| No | 684 (84.1%) | 125 (85.0%) | 115 (86.5%) |
| Yes | 129 (15.9%) | 22 (15.0%) | 18 (13.5%) |
| **Both parents atopic disease (eczema, asthma, hay fever)** |  | | |
| No | 558 (68.6%) | 104 (70.7%) | 95 (71.4%) |
| Yes | 255 (31.4%) | 43 (29.3%) | 38 (28.6%) |
| **Smoking in household during pregnancy** |  | | |
| No | 695 (85.5%) | 127 (86.4%) | 115 (86.5%) |
| Yes | 118 (14.5%) | 20 (13.6%) | 18 (13.5%) |
| **Family has animals (cat, dog, livestock)** |  | | |
| No | 374 (46.0%) | 66 (44.9%) | 62 (46.6%) |
| Yes | 439 (54.0%) | 81 (55.1%) | 71 (53.4%) |
| **Number of household inhabitants** |  | | |
| 1/2 | 390 (48.0%) | 65 (44.2%) | 57 (42.9%) |
| 3/4 | 369 (45.4%) | 77 (52.4%) | 71 (53.4%) |
| ≥5 | 54 (6.6%) | 5 (3.4%) | 5 (3.8%) |
| **Number of siblings at time of birth** |  | | |
| No siblings | 437 (53.8%) | 67 (45.6%) | 61 (45.9%) |
| 1 sibling | 251 (30.9%) | 53 (36.1%) | 48 (36.1%) |
| ≥2 siblings | 125 (15.4%) | 27 (18.4%) | 24 (18.0%) |
| **Number of household inhabitants of school age** |  | | |
| 0 | 687 (84.5%) | 123 (83.7%) | 111 (83.5%) |
| 1 | 83 (10.2%) | 16 (10.9%) | 14 (10.5%) |
| ≥2 | 43 (5.3%) | 8 (5.4%) | 8 (6.0%) |
| **Maternal antibiotic use in 3rd trimester (excl. labour)** |  | | |
| No | 709 (87.2%) | 127 (86.4%) | 117 (88.0%) |
| Yes | 104 (12.8%) | 20 (13.6%) | 16 (12.0%) |
| **Maternal probiotic use during pregnancy** |  | | |
| No | 669 (82.3%) | 119 (81.0%) | 107 (80.5%) |
| Yes | 144 (17.7%) | 28 (19.0%) | 26 (19.5%) |
| **Maternal vitamin D during pregnancy** |  | | |
| No | 262 (32.2%) | 43 (29.3%) | 40 (30.1%) |
| Yes | 551 (67.8%) | 104 (70.7%) | 93 (69.9%) |
| **Mother GBS positive during pregnancy** |  | | |
| No | 666 (81.9%) | 125 (85.0%) | 111 (83.5%) |
| Yes | 147 (18.1%) | 22 (15.0%) | 22 (16.5%) |
| **Maternal antibiotics during labour** |  | | |
| No | 624 (76.8%) | 113 (76.9%) | 101 (75.9%) |
| Yes | 189 (23.2%) | 34 (23.1%) | 32 (24.1%) |
| **Method of birth** |  | | |
| C-section | 296 (36.4%) | 53 (36.1%) | 47 (35.3%) |
| Vaginal | 517 (63.6%) | 94 (63.9%) | 86 (64.7%) |
| **Birth weight (g)** |  | | |
| median (IQR) | 3380.0 (660.0) | 3535.0 (580.0) | 3540.0 (510.0) |
| **Gestational age (wk)** |  | | |
| median (IQR) | 39.3 (2.0) | 39.3 (2.0) | 39.4 (2.0) |
| **Maternal age (y) at birth** |  | | |
| median (IQR) | 33.0 (5.8) | 33.6 (5.6) | 33.9 (5.5) |
| **Season of birth** |  | | |
| Summer | 194 (23.9%) | 37 (25.2%) | 33 (24.8%) |
| Autumn | 225 (27.7%) | 35 (23.8%) | 34 (25.6%) |
| Winter | 194 (23.9%) | 40 (27.2%) | 36 (27.1%) |
| Spring | 200 (24.6%) | 35 (23.8%) | 30 (22.6%) |
| **Vitamin D supplement in hospital** |  | | |
| No | 572 (70.4%) | 116 (78.9%) | 104 (78.2%) |
| Yes | 241 (29.6%) | 31 (21.1%) | 29 (21.8%) |
| **Birth order** |  | | |
| 1 | 453 (55.7%) | 71 (48.3%) | 64 (48.1%) |
| 2 | 246 (30.3%) | 51 (34.7%) | 46 (34.6%) |
| 3 | 95 (11.7%) | 21 (14.3%) | 19 (14.3%) |
| 4 | 15 (1.8%) | 3 (2.0%) | 3 (2.3%) |
| 5 | 4 (0.5%) | 1 (0.7%) | 1 (0.8%) |
| **Daycare attendance for participant during first year of life** |  | | |
| No | 507 (62.4%) | 87 (59.2%) | 79 (59.4%) |
| Yes | 306 (37.6%) | 60 (40.8%) | 54 (40.6%) |
| **Breastfeeding** |  | | |
| None to ≤3m | 162 (19.9%) | 19 (12.9%) | 17 (12.8%) |
| >3m | 651 (80.1%) | 128 (87.1%) | 116 (87.2%) |

**Supplementary Table 2. Tabulated odds of topical steroid treatment and eczema severity by six months**

| Eczema severity by 6m | Topical steroid treatment by 6m | | Odd ratio (95%CI) |
| --- | --- | --- | --- |
|  | No  n (%) | Yes  n (%) |  |
| Clear / Mild | 120 | 90 | 1.0 ref |
| Moderate | 42 | 59 | 1.87 (1.2-3.1)* |
| Severe | 3 | 16 | 7.11 (1.9-26.1)** |

*p<0.05 **p<0.001

**Supplementary Table 3. Elastic-net stability selection frequencies, FS — cytokines + a priori clinical.***

| Variable | Times selected | Selection frequency | Stability band |
| --- | --- | --- | --- |
| **LPS: IL-10** | **2,381** | **0.952** | **≥ 0.90** |
| ***H. influenzae*: IL-1Ra** | **2,346** | **0.938** | **≥ 0.90** |
| **PEPG: IFN-γ** | **2,302** | **0.921** | **≥ 0.90** |
| ***E. coli*: MCP-1** | **2,253** | **0.901** | **≥ 0.90** |
| ***S. aureus*: MIP-1α** | **2,116** | **0.846** | **0.80–0.89** |
| ***S. pyogenes*: IL-1Ra** | **2,083** | **0.833** | **0.80–0.89** |
| **Pam3CSK4: IL-6** | **2,011** | **0.804** | **0.80–0.89** |
| **Pam3CSK4: MIP-1α** | **1,998** | **0.799** | **0.75–0.79** |
| **Pam3CSK4: IL-1Ra** | **1,968** | **0.787** | **0.75–0.79** |
| **LPS: IL-1β** | **1,940** | **0.776** | **0.75–0.79** |
| ***L. monocytogenes*: IL-1β** | **1,934** | **0.774** | **0.75–0.79** |
| ***S. pneumoniae*: IP-10** | **1,924** | **0.770** | **0.75–0.79** |
| **Pam3CSK4: IP-10** | **1,921** | **0.768** | **0.75–0.79** |
| *L. monocytogenes*: IP-10 | 1,833 | 0.733 | 0.70–0.74 |
| *S. pneumoniae*: TNF-α | 1,746 | 0.698 | 0.60–0.69 |
| *C. albicans*: MCP-1 | 1,721 | 0.688 | 0.60–0.69 |
| LPS: IP-10 | 1,697 | 0.679 | 0.60–0.69 |
| *C. albicans*: TNF-α | 1,666 | 0.666 | 0.60–0.69 |
| *S. pneumoniae*: IFN-γ | 1,661 | 0.664 | 0.60–0.69 |
| *S. aureus*: MCP-1 | 1,616 | 0.646 | 0.60–0.69 |
| R848: IP-10 | 1,593 | 0.637 | 0.60–0.69 |
| R848: MCP-1 | 1,588 | 0.635 | 0.60–0.69 |
| *E. coli*: IL-1Ra | 1,576 | 0.630 | 0.60–0.69 |
| Pam3CSK4: IFN-γ | 1,541 | 0.616 | 0.60–0.69 |
| R848: IL-1β | 1,540 | 0.616 | 0.60–0.69 |
| *S. pneumoniae*: IL-8 | 1,530 | 0.612 | 0.60–0.69 |
| *L. monocytogenes*: IFN-γ | 1,500 | 0.600 | 0.60–0.69 |
| Pam3CSK4: MIP-1β | 1,488 | 0.595 | < 0.60 |
| *H. influenzae*: IL-10 | 1,483 | 0.593 | < 0.60 |
| *S. aureus*: IFN-γ | 1,472 | 0.589 | < 0.60 |
| *L. monocytogenes*: IL-1Ra | 1,464 | 0.586 | < 0.60 |
| *S. pyogenes*: IFN-γ | 1,441 | 0.576 | < 0.60 |
| R848: IL-10 | 1,430 | 0.572 | < 0.60 |
| *E. coli*: IL-10 | 1,410 | 0.564 | < 0.60 |
| LPS: IL-1Ra | 1,403 | 0.561 | < 0.60 |
| *S. pyogenes*: MCP-1 | 1,398 | 0.559 | < 0.60 |
| *C. albicans*: IFN-γ | 1,391 | 0.556 | < 0.60 |
| Pam3CSK4: IL-1β | 1,376 | 0.550 | < 0.60 |
| *S. pyogenes*: IP-10 | 1,375 | 0.550 | < 0.60 |
| PEPG: IP-10 | 1,375 | 0.550 | < 0.60 |
| *H. influenzae*: MIP-1α | 1,374 | 0.550 | < 0.60 |
| *L. monocytogenes*: MCP-1 | 1,372 | 0.549 | < 0.60 |
| R848: IL-1Ra | 1,339 | 0.536 | < 0.60 |
| R848: MIP-1β | 1,320 | 0.528 | < 0.60 |
| *E. coli*: IP-10 | 1,319 | 0.528 | < 0.60 |
| *S. pyogenes*: IL-1β | 1,315 | 0.526 | < 0.60 |
| *S. pyogenes*: MIP-1α | 1,309 | 0.524 | < 0.60 |
| *S. pneumoniae*: MCP-1 | 1,298 | 0.519 | < 0.60 |
| *S. aureus*: IL-1β | 1,296 | 0.518 | < 0.60 |
| *E. coli*: IFN-γ | 1,294 | 0.518 | < 0.60 |
| *S. pneumoniae*: IL-1Ra | 1,290 | 0.516 | < 0.60 |
| *S. pneumoniae*: MIP-1α | 1,274 | 0.510 | < 0.60 |
| *S. pyogenes*: IL-8 | 1,271 | 0.508 | < 0.60 |
| *C. albicans*: IP-10 | 1,267 | 0.507 | < 0.60 |
| *C. albicans*: IL-10 | 1,257 | 0.503 | < 0.60 |
| LPS: IFN-γ | 1,243 | 0.497 | < 0.60 |
| *S. aureus*: IP-10 | 1,232 | 0.493 | < 0.60 |
| *H. influenzae*: MCP-1 | 1,229 | 0.492 | < 0.60 |
| *C. albicans*: IL-1β | 1,212 | 0.485 | < 0.60 |
| PEPG: MCP-1 | 1,172 | 0.469 | < 0.60 |
| R848: IFN-γ | 1,166 | 0.466 | < 0.60 |
| *S. aureus*: IL-1Ra | 1,147 | 0.459 | < 0.60 |
| PEPG: MIP-1β | 1,144 | 0.458 | < 0.60 |
| *H. influenzae*: IP-10 | 1,142 | 0.457 | < 0.60 |
| PEPG: MIG | 1,141 | 0.456 | < 0.60 |
| PEPG: TNF-α | 1,131 | 0.452 | < 0.60 |
| *E. coli*: MIF | 1,122 | 0.449 | < 0.60 |
| *L. monocytogenes*: TNF-α | 1,122 | 0.449 | < 0.60 |
| *S. pyogenes*: IL-10 | 1,120 | 0.448 | < 0.60 |
| *L. monocytogenes*: IL-10 | 1,119 | 0.448 | < 0.60 |
| *S. pyogenes*: MIP-1β | 1,108 | 0.443 | < 0.60 |
| *S. pneumoniae*: IL-1β | 1,103 | 0.441 | < 0.60 |
| *E. coli*: MIP-1β | 1,086 | 0.434 | < 0.60 |
| *S. aureus*: MIP-1β | 1,084 | 0.434 | < 0.60 |
| *E. coli*: IL-1β | 1,076 | 0.430 | < 0.60 |
| *S. pyogenes*: TNF-α | 1,076 | 0.430 | < 0.60 |
| *H. influenzae*: IL-8 | 1,075 | 0.430 | < 0.60 |
| PEPG: MIP-1α | 1,075 | 0.430 | < 0.60 |
| *S. pneumoniae*: IL-6 | 1,067 | 0.427 | < 0.60 |
| R848: IL-8 | 1,064 | 0.426 | < 0.60 |
| LPS: MIP-1α | 1,051 | 0.420 | < 0.60 |
| *S. aureus*: IL-8 | 1,038 | 0.415 | < 0.60 |
| Pam3CSK4: TNF-α | 1,030 | 0.412 | < 0.60 |
| *H. influenzae*: IFN-γ | 1,029 | 0.412 | < 0.60 |
| LPS: MCP-1 | 1,016 | 0.406 | < 0.60 |
| *S. pyogenes*: IL-6 | 1,007 | 0.403 | < 0.60 |
| PEPG: IL-1β | 1,005 | 0.402 | < 0.60 |
| *C. albicans*: MIP-1α | 1,000 | 0.400 | < 0.60 |
| *L. monocytogenes*: MIP-1β | 997 | 0.399 | < 0.60 |
| *S. pneumoniae*: MIP-1β | 994 | 0.398 | < 0.60 |
| PEPG: IL-10 | 982 | 0.393 | < 0.60 |
| *S. pneumoniae*: IL-10 | 976 | 0.390 | < 0.60 |
| R848: IL-6 | 961 | 0.384 | < 0.60 |
| *S. aureus*: IL-10 | 956 | 0.382 | < 0.60 |
| *S. aureus*: IL-6 | 954 | 0.382 | < 0.60 |
| PEPG: MIF | 952 | 0.381 | < 0.60 |
| R848: TNF-α | 935 | 0.374 | < 0.60 |
| *C. albicans*: IL-6 | 923 | 0.369 | < 0.60 |
| *C. albicans*: IL-1Ra | 908 | 0.363 | < 0.60 |
| *E. coli*: MIG | 907 | 0.363 | < 0.60 |
| R848: MIP-1α | 900 | 0.360 | < 0.60 |
| Pam3CSK4: IL-8 | 886 | 0.354 | < 0.60 |
| *S. aureus*: TNF-α | 886 | 0.354 | < 0.60 |
| LPS: IL-8 | 868 | 0.347 | < 0.60 |
| *L. monocytogenes*: IL-8 | 861 | 0.344 | < 0.60 |
| PEPG: IL-8 | 856 | 0.342 | < 0.60 |
| *C. albicans*: IL-8 | 852 | 0.341 | < 0.60 |
| *E. coli*: IL-8 | 852 | 0.341 | < 0.60 |
| Pam3CSK4: IL-10 | 810 | 0.324 | < 0.60 |
| PEPG: IL-1Ra | 800 | 0.320 | < 0.60 |
| LPS: TNF-α | 790 | 0.316 | < 0.60 |
| *L. monocytogenes*: IL-6 | 784 | 0.314 | < 0.60 |
| Pam3CSK4: MCP-1 | 775 | 0.310 | < 0.60 |
| *L. monocytogenes*: MIP-1α | 773 | 0.309 | < 0.60 |
| PEPG: IL-6 | 771 | 0.308 | < 0.60 |
| LPS: MIP-1β | 733 | 0.293 | < 0.60 |
| *H. influenzae*: IL-1β | 725 | 0.290 | < 0.60 |
| *H. influenzae*: IL-6 | 719 | 0.288 | < 0.60 |
| LPS: IL-6 | 693 | 0.277 | < 0.60 |
| *E. coli*: IL-6 | 660 | 0.264 | < 0.60 |
| *C. albicans*: MIP-1β | 614 | 0.246 | < 0.60 |
| *H. influenzae*: TNF-α | 589 | 0.236 | < 0.60 |
| *E. coli*: TNF-α | 563 | 0.225 | < 0.60 |
| *E. coli*: MIP-1α | 560 | 0.224 | < 0.60 |
| *H. influenzae*: MIP-1β | 476 | 0.190 | < 0.60 |

*Frequency is the proportion of successful ENR fits (n = 2,500) in which each variable was retained. Variables in bold meet the pre-specified stability threshold of 0.75.

**Supplementary Table 4. Elastic-net stability selection frequencies, FS — cytokines + full clinical.***

| Variable | Times selected | Selection frequency | Stability band |
| --- | --- | --- | --- |
| **Baby: Eczema by 6m** | **2,469** | **0.988** | **≥ 0.90** |
| **Baby: Topical steroid treatment by 6m** | **2,425** | **0.970** | **≥ 0.90** |
| **Mother: University level education** | **2,226** | **0.890** | **0.80–0.89** |
| **Baby: Daycare attendance** | **2,223** | **0.889** | **0.80–0.89** |
| **Mother: Antibiotics last trimester pregnancy** | **2,192** | **0.877** | **0.80–0.89** |
| ***S. pyogenes*: IL-1Ra** | **2,184** | **0.874** | **0.80–0.89** |
| **PEPG: IFN-γ** | **2,040** | **0.816** | **0.80–0.89** |
| **Pam3CSK4: MIP-1α** | **1,981** | **0.792** | **0.75–0.79** |
| **Baby: Birth weight** | **1,952** | **0.781** | **0.75–0.79** |
| ***H. influenzae*: IL-1Ra** | **1,946** | **0.778** | **0.75–0.79** |
| **Household: Cat/Dog/Livestock** | **1,937** | **0.775** | **0.75–0.79** |
| Baby: Birth season | 1,858 | 0.743 | 0.70–0.74 |
| LPS: IL-10 | 1,855 | 0.742 | 0.70–0.74 |
| Pam3CSK4: IL-6 | 1,841 | 0.736 | 0.70–0.74 |
| *S. pyogenes*: IFN-γ | 1,786 | 0.714 | 0.70–0.74 |
| LPS: IL-1β | 1,756 | 0.702 | 0.70–0.74 |
| *H. influenzae*: IL-10 | 1,731 | 0.692 | 0.60–0.69 |
| Pam3CSK4: IL-1Ra | 1,678 | 0.671 | 0.60–0.69 |
| Household: 1 sibling | 1,641 | 0.656 | 0.60–0.69 |
| R848: IP-10 | 1,618 | 0.647 | 0.60–0.69 |
| *E. coli*: MCP-1 | 1,607 | 0.643 | 0.60–0.69 |
| LPS: IL-1Ra | 1,574 | 0.630 | 0.60–0.69 |
| Baby: BCG | 1,571 | 0.628 | 0.60–0.69 |
| *E. coli*: IL-1Ra | 1,549 | 0.620 | 0.60–0.69 |
| *L. monocytogenes*: IL-1β | 1,549 | 0.620 | 0.60–0.69 |
| LPS: IP-10 | 1,543 | 0.617 | 0.60–0.69 |
| *S. aureus*: MIP-1α | 1,534 | 0.614 | 0.60–0.69 |
| R848: MCP-1 | 1,441 | 0.576 | < 0.60 |
| Pam3CSK4: IP-10 | 1,435 | 0.574 | < 0.60 |
| Household: Parent(s) ANY atopic disease | 1,433 | 0.573 | < 0.60 |
| *S. pneumoniae*: IP-10 | 1,402 | 0.561 | < 0.60 |
| R848: IL-1β | 1,394 | 0.558 | < 0.60 |
| Mother: Probiotics during pregnancy | 1,392 | 0.557 | < 0.60 |
| Household: History food allergy | 1,387 | 0.555 | < 0.60 |
| R848: IL-1Ra | 1,372 | 0.549 | < 0.60 |
| Baby: Birth order | 1,366 | 0.546 | < 0.60 |
| Household: Parent(s) born Southeast Asia | 1,348 | 0.539 | < 0.60 |
| *S. aureus*: MCP-1 | 1,343 | 0.537 | < 0.60 |
| R848: IFN-γ | 1,330 | 0.532 | < 0.60 |
| Pam3CSK4: IFN-γ | 1,328 | 0.531 | < 0.60 |
| *S. pneumoniae*: TNF-α | 1,327 | 0.531 | < 0.60 |
| Baby: Caesarean section | 1,310 | 0.524 | < 0.60 |
| Baby: Vaginal delivery | 1,310 | 0.524 | < 0.60 |
| *L. monocytogenes*: IFN-γ | 1,302 | 0.521 | < 0.60 |
| Baby: Vitamin D in hospital | 1,298 | 0.519 | < 0.60 |
| Mother: Age at participant's birth | 1,281 | 0.512 | < 0.60 |
| Baby: Male | 1,256 | 0.502 | < 0.60 |
| Mother: Antibiotics during labour | 1,251 | 0.500 | < 0.60 |
| Mother: Vitamin D during pregnancy | 1,220 | 0.488 | < 0.60 |
| *C. albicans*: IL-10 | 1,217 | 0.487 | < 0.60 |
| *E. coli*: MIF | 1,154 | 0.462 | < 0.60 |
| *E. coli*: IL-1β | 1,152 | 0.461 | < 0.60 |
| *S. pyogenes*: MIP-1α | 1,147 | 0.459 | < 0.60 |
| *C. albicans*: MCP-1 | 1,146 | 0.458 | < 0.60 |
| *L. monocytogenes*: IP-10 | 1,141 | 0.456 | < 0.60 |
| PEPG: IP-10 | 1,136 | 0.454 | < 0.60 |
| *L. monocytogenes*: MCP-1 | 1,119 | 0.448 | < 0.60 |
| PEPG: MIG | 1,111 | 0.444 | < 0.60 |
| *E. coli*: IL-10 | 1,110 | 0.444 | < 0.60 |
| Pam3CSK4: MIP-1β | 1,110 | 0.444 | < 0.60 |
| *C. albicans*: IFN-γ | 1,097 | 0.439 | < 0.60 |
| *S. pneumoniae*: IFN-γ | 1,093 | 0.437 | < 0.60 |
| *S. pyogenes*: IL-1β | 1,090 | 0.436 | < 0.60 |
| *C. albicans*: IL-1β | 1,089 | 0.436 | < 0.60 |
| Household: Smokers lived in house during pregnancy | 1,086 | 0.434 | < 0.60 |
| *S. pneumoniae*: IL-8 | 1,070 | 0.428 | < 0.60 |
| *L. monocytogenes*: IL-1Ra | 1,063 | 0.425 | < 0.60 |
| *S. aureus*: IP-10 | 1,058 | 0.423 | < 0.60 |
| Baby: Gestational age | 1,057 | 0.423 | < 0.60 |
| *S. pyogenes*: IL-10 | 1,053 | 0.421 | < 0.60 |
| *C. albicans*: TNF-α | 1,049 | 0.420 | < 0.60 |
| Breastfeeding >3 months | 1,047 | 0.419 | < 0.60 |
| *H. influenzae*: MCP-1 | 1,035 | 0.414 | < 0.60 |
| *S. aureus*: IL-1β | 1,034 | 0.414 | < 0.60 |
| *E. coli*: IFN-γ | 1,000 | 0.400 | < 0.60 |
| *L. monocytogenes*: IL-10 | 999 | 0.400 | < 0.60 |
| *S. pyogenes*: IP-10 | 992 | 0.397 | < 0.60 |
| *S. pyogenes*: MCP-1 | 986 | 0.394 | < 0.60 |
| *C. albicans*: IP-10 | 979 | 0.392 | < 0.60 |
| *E. coli*: IP-10 | 973 | 0.389 | < 0.60 |
| LPS: IFN-γ | 970 | 0.388 | < 0.60 |
| Pam3CSK4: TNF-α | 965 | 0.386 | < 0.60 |
| *S. aureus*: IL-10 | 958 | 0.383 | < 0.60 |
| *S. pneumoniae*: MCP-1 | 958 | 0.383 | < 0.60 |
| Pam3CSK4: IL-8 | 955 | 0.382 | < 0.60 |
| *S. aureus*: IFN-γ | 953 | 0.381 | < 0.60 |
| *S. pneumoniae*: IL-1β | 949 | 0.380 | < 0.60 |
| *S. pneumoniae*: IL-10 | 947 | 0.379 | < 0.60 |
| 2 or more siblings | 942 | 0.377 | < 0.60 |
| *S. pneumoniae*: IL-6 | 936 | 0.374 | < 0.60 |
| *S. pneumoniae*: MIP-1α | 935 | 0.374 | < 0.60 |
| *S. pneumoniae*: IL-1Ra | 924 | 0.370 | < 0.60 |
| Pam3CSK4: IL-1β | 918 | 0.367 | < 0.60 |
| *E. coli*: MIP-1β | 905 | 0.362 | < 0.60 |
| *S. aureus*: IL-8 | 888 | 0.355 | < 0.60 |
| *S. aureus*: MIP-1β | 859 | 0.344 | < 0.60 |
| *S. pyogenes*: MIP-1β | 856 | 0.342 | < 0.60 |
| *S. pyogenes*: IL-8 | 853 | 0.341 | < 0.60 |
| *S. pneumoniae*: MIP-1β | 836 | 0.334 | < 0.60 |
| *L. monocytogenes*: MIP-1β | 833 | 0.333 | < 0.60 |
| R848: MIP-1β | 822 | 0.329 | < 0.60 |
| *L. monocytogenes*: TNF-α | 819 | 0.328 | < 0.60 |
| *S. pyogenes*: TNF-α | 809 | 0.324 | < 0.60 |
| *H. influenzae*: IP-10 | 797 | 0.319 | < 0.60 |
| Pam3CSK4: MCP-1 | 797 | 0.319 | < 0.60 |
| *C. albicans*: IL-6 | 790 | 0.316 | < 0.60 |
| *E. coli*: IL-8 | 782 | 0.313 | < 0.60 |
| *H. influenzae*: IL-1β | 769 | 0.308 | < 0.60 |
| *L. monocytogenes*: MIP-1α | 768 | 0.307 | < 0.60 |
| LPS: MIP-1α | 758 | 0.303 | < 0.60 |
| *E. coli*: MIG | 745 | 0.298 | < 0.60 |
| *C. albicans*: MIP-1α | 743 | 0.297 | < 0.60 |
| *H. influenzae*: IL-8 | 735 | 0.294 | < 0.60 |
| *C. albicans*: IL-1Ra | 732 | 0.293 | < 0.60 |
| *L. monocytogenes*: IL-8 | 729 | 0.292 | < 0.60 |
| LPS: MCP-1 | 726 | 0.290 | < 0.60 |
| R848: IL-8 | 726 | 0.290 | < 0.60 |
| *S. aureus*: IL-1Ra | 726 | 0.290 | < 0.60 |
| LPS: MIP-1β | 711 | 0.284 | < 0.60 |
| *S. pyogenes*: IL-6 | 709 | 0.284 | < 0.60 |
| *S. aureus*: IL-6 | 709 | 0.284 | < 0.60 |
| PEPG: MIP-1α | 704 | 0.282 | < 0.60 |
| PEPG: MIP-1β | 702 | 0.281 | < 0.60 |
| Pam3CSK4: IL-10 | 689 | 0.276 | < 0.60 |
| *H. influenzae*: IFN-γ | 686 | 0.274 | < 0.60 |
| R848: IL-6 | 683 | 0.273 | < 0.60 |
| PEPG: TNF-α | 673 | 0.269 | < 0.60 |
| *L. monocytogenes*: IL-6 | 651 | 0.260 | < 0.60 |
| PEPG: IL-8 | 648 | 0.259 | < 0.60 |
| *S. aureus*: TNF-α | 634 | 0.254 | < 0.60 |
| *C. albicans*: IL-8 | 629 | 0.252 | < 0.60 |
| PEPG: MIF | 619 | 0.248 | < 0.60 |
| PEPG: IL-10 | 608 | 0.243 | < 0.60 |
| *C. albicans*: MIP-1β | 607 | 0.243 | < 0.60 |
| PEPG: IL-1β | 597 | 0.239 | < 0.60 |
| LPS: IL-8 | 568 | 0.227 | < 0.60 |
| PEPG: IL-1Ra | 542 | 0.217 | < 0.60 |
| R848: IL-10 | 530 | 0.212 | < 0.60 |
| LPS: IL-6 | 499 | 0.200 | < 0.60 |
| *E. coli*: MIP-1α | 495 | 0.198 | < 0.60 |
| R848: TNF-α | 462 | 0.185 | < 0.60 |
| *E. coli*: IL-6 | 446 | 0.178 | < 0.60 |
| R848: MIP-1α | 446 | 0.178 | < 0.60 |
| PEPG: IL-6 | 439 | 0.176 | < 0.60 |
| PEPG: MCP-1 | 434 | 0.174 | < 0.60 |
| *H. influenzae*: MIP-1β | 382 | 0.153 | < 0.60 |
| *H. influenzae*: MIP-1α | 372 | 0.149 | < 0.60 |
| *H. influenzae*: IL-6 | 364 | 0.146 | < 0.60 |
| LPS: TNF-α | 342 | 0.137 | < 0.60 |
| *H. influenzae*: TNF-α | 321 | 0.128 | < 0.60 |
| *E. coli*: TNF-α | 272 | 0.109 | < 0.60 |

*Frequency is the proportion of successful ENR fits (n = 2,500) in which each variable was retained. Variables in bold meet the pre-specified stability threshold of 0.75.

**Supplementary Table 5. Elastic-net stability selection frequencies, FA — cytokines + a priori clinical.***

| Variable | Times selected | Selection frequency | Stability band |
| --- | --- | --- | --- |
| ***E. coli*: MCP-1** | **2,264** | **0.909** | **≥ 0.90** |
| **S*. pyogenes*: IP-10** | **2,162** | **0.868** | **0.80–0.89** |
| **Pam3CSK4: IL-1Ra** | **2,072** | **0.832** | **0.80–0.89** |
| ***H. influenzae*: IL-1Ra** | **1,949** | **0.782** | **0.75–0.79** |
| PEPG: IFN-γ | 1,819 | 0.730 | 0.70–0.74 |
| *S. aureus*: IFN-γ | 1,800 | 0.723 | 0.70–0.74 |
| R848: IL-1Ra | 1,794 | 0.720 | 0.70–0.74 |
| R848: IP-10 | 1,755 | 0.705 | 0.70–0.74 |
| LPS: MCP-1 | 1,746 | 0.701 | 0.70–0.74 |
| *S. pneumoniae*: MIP-1α | 1,724 | 0.692 | 0.60–0.69 |
| *S. aureus*: IL-1β | 1,656 | 0.665 | 0.60–0.69 |
| LPS: IL-10 | 1,650 | 0.662 | 0.60–0.69 |
| *H. influenzae*: MIP-1β | 1,588 | 0.637 | 0.60–0.69 |
| R848: IL-1β | 1,578 | 0.633 | 0.60–0.69 |
| *H. influenzae*: MIP-1α | 1,563 | 0.627 | 0.60–0.69 |
| *H. influenzae*: IP-10 | 1,558 | 0.625 | 0.60–0.69 |
| LPS: IL-1β | 1,507 | 0.605 | 0.60–0.69 |
| *S. pneumoniae*: IFN-γ | 1,471 | 0.591 | < 0.60 |
| LPS: MIP-1α | 1,446 | 0.581 | < 0.60 |
| *E. coli*: MIF | 1,431 | 0.575 | < 0.60 |
| *E. coli*: MIP-1β | 1,430 | 0.574 | < 0.60 |
| Pam3CSK4: MIP-1α | 1,426 | 0.573 | < 0.60 |
| *S. pneumoniae*: IP-10 | 1,402 | 0.563 | < 0.60 |
| *L. monocytogenes*: IL-1β | 1,393 | 0.559 | < 0.60 |
| *S. pneumoniae*: IL-8 | 1,364 | 0.548 | < 0.60 |
| *S. pyogenes*: MCP-1 | 1,360 | 0.546 | < 0.60 |
| *C. albicans*: IFN-γ | 1,353 | 0.543 | < 0.60 |
| *E. coli*: IP-10 | 1,350 | 0.542 | < 0.60 |
| PEPG: MIP-1α | 1,302 | 0.523 | < 0.60 |
| Pam3CSK4: IP-10 | 1,298 | 0.521 | < 0.60 |
| PEPG: MCP-1 | 1,280 | 0.514 | < 0.60 |
| *S. aureus*: MCP-1 | 1,276 | 0.512 | < 0.60 |
| *S. pneumoniae*: IL-1Ra | 1,271 | 0.510 | < 0.60 |
| *L. monocytogenes*: IP-10 | 1,205 | 0.484 | < 0.60 |
| *S. pyogenes*: IL-1Ra | 1,197 | 0.480 | < 0.60 |
| *L. monocytogenes*: IL-1Ra | 1,183 | 0.475 | < 0.60 |
| *H. influenzae*: MCP-1 | 1,182 | 0.474 | < 0.60 |
| *S. pneumoniae*: TNF-α | 1,178 | 0.473 | < 0.60 |
| *C. albicans*: MCP-1 | 1,166 | 0.468 | < 0.60 |
| Pam3CSK4: IL-1β | 1,165 | 0.468 | < 0.60 |
| *L. monocytogenes*: MCP-1 | 1,163 | 0.467 | < 0.60 |
| *C. albicans*: TNF-α | 1,151 | 0.462 | < 0.60 |
| *L. monocytogenes*: IL-10 | 1,144 | 0.459 | < 0.60 |
| Pam3CSK4: IL-6 | 1,139 | 0.457 | < 0.60 |
| *S. pyogenes*: IL-8 | 1,137 | 0.456 | < 0.60 |
| LPS: IL-1Ra | 1,137 | 0.456 | < 0.60 |
| PEPG: IP-10 | 1,136 | 0.456 | < 0.60 |
| *H. influenzae*: IL-6 | 1,129 | 0.453 | < 0.60 |
| PEPG: IL-1β | 1,104 | 0.443 | < 0.60 |
| *E. coli*: IL-1Ra | 1,102 | 0.442 | < 0.60 |
| *S. aureus*: IL-6 | 1,099 | 0.441 | < 0.60 |
| *S. pyogenes*: IL-10 | 1,087 | 0.436 | < 0.60 |
| PEPG: IL-6 | 1,079 | 0.433 | < 0.60 |
| *S. aureus*: IL-10 | 1,074 | 0.431 | < 0.60 |
| R848: IL-10 | 1,069 | 0.429 | < 0.60 |
| Pam3CSK4: IFN-γ | 1,066 | 0.428 | < 0.60 |
| *S. pneumoniae*: MCP-1 | 1,034 | 0.415 | < 0.60 |
| *S. pyogenes*: IFN-γ | 1,026 | 0.412 | < 0.60 |
| *S. pneumoniae*: MIP-1β | 1,000 | 0.401 | < 0.60 |
| *L. monocytogenes*: IFN-γ | 983 | 0.395 | < 0.60 |
| *H. influenzae*: IFN-γ | 971 | 0.390 | < 0.60 |
| PEPG: MIG | 961 | 0.386 | < 0.60 |
| *S. aureus*: IP-10 | 950 | 0.381 | < 0.60 |
| LPS: TNF-α | 934 | 0.375 | < 0.60 |
| *S. pyogenes*: IL-1β | 933 | 0.374 | < 0.60 |
| *S. pyogenes*: TNF-α | 930 | 0.373 | < 0.60 |
| *S. aureus*: MIP-1α | 891 | 0.358 | < 0.60 |
| *C. albicans*: IL-1β | 887 | 0.356 | < 0.60 |
| *C. albicans*: IL-10 | 884 | 0.355 | < 0.60 |
| LPS: IP-10 | 870 | 0.349 | < 0.60 |
| *S. pneumoniae*: IL-1β | 846 | 0.340 | < 0.60 |
| *S. pyogenes*: MIP-1α | 837 | 0.336 | < 0.60 |
| *S. pyogenes*: MIP-1β | 833 | 0.334 | < 0.60 |
| LPS: IFN-γ | 817 | 0.328 | < 0.60 |
| *C. albicans*: IP-10 | 814 | 0.327 | < 0.60 |
| LPS: IL-8 | 795 | 0.319 | < 0.60 |
| *S. pneumoniae*: IL-10 | 785 | 0.315 | < 0.60 |
| *L. monocytogenes*: MIP-1α | 784 | 0.315 | < 0.60 |
| *S. aureus*: IL-8 | 779 | 0.313 | < 0.60 |
| *S. pneumoniae*: IL-6 | 761 | 0.305 | < 0.60 |
| *C. albicans*: IL-1Ra | 760 | 0.305 | < 0.60 |
| *C. albicans*: IL-8 | 759 | 0.305 | < 0.60 |
| *E. coli*: MIP-1α | 757 | 0.304 | < 0.60 |
| *S. pyogenes*: IL-6 | 757 | 0.304 | < 0.60 |
| *L. monocytogenes*: IL-8 | 750 | 0.301 | < 0.60 |
| *E. coli*: MIG | 734 | 0.295 | < 0.60 |
| R848: MIP-1β | 709 | 0.285 | < 0.60 |
| *L. monocytogenes*: TNF-α | 698 | 0.280 | < 0.60 |
| *E. coli*: IL-1β | 694 | 0.279 | < 0.60 |
| PEPG: MIF | 690 | 0.277 | < 0.60 |
| R848: IFN-γ | 690 | 0.277 | < 0.60 |
| *L. monocytogenes*: IL-6 | 678 | 0.272 | < 0.60 |
| R848: IL-6 | 664 | 0.267 | < 0.60 |
| *C. albicans*: MIP-1α | 651 | 0.261 | < 0.60 |
| R848: MCP-1 | 650 | 0.261 | < 0.60 |
| *E. coli*: IFN-γ | 644 | 0.259 | < 0.60 |
| PEPG: IL-10 | 627 | 0.252 | < 0.60 |
| *L. monocytogenes*: MIP-1β | 620 | 0.249 | < 0.60 |
| *C. albicans*: MIP-1β | 619 | 0.248 | < 0.60 |
| Pam3CSK4: MCP-1 | 619 | 0.248 | < 0.60 |
| *E. coli*: IL-8 | 596 | 0.239 | < 0.60 |
| R848: TNF-α | 593 | 0.238 | < 0.60 |
| Pam3CSK4: MIP-1β | 585 | 0.235 | < 0.60 |
| PEPG: TNF-α | 578 | 0.232 | < 0.60 |
| *S. aureus*: IL-1Ra | 575 | 0.231 | < 0.60 |
| Pam3CSK4: TNF-α | 511 | 0.205 | < 0.60 |
| Pam3CSK4: IL-8 | 501 | 0.201 | < 0.60 |
| Pam3CSK4: IL-10 | 495 | 0.199 | < 0.60 |
| LPS: MIP-1β | 487 | 0.196 | < 0.60 |
| *H. influenzae*: IL-1β | 485 | 0.195 | < 0.60 |
| *H. influenzae*: TNF-α | 483 | 0.194 | < 0.60 |
| LPS: IL-6 | 430 | 0.173 | < 0.60 |
| R848: IL-8 | 399 | 0.160 | < 0.60 |
| *C. albicans*: IL-6 | 394 | 0.158 | < 0.60 |
| PEPG: MIP-1β | 369 | 0.148 | < 0.60 |
| *H. influenzae*: IL-8 | 368 | 0.148 | < 0.60 |
| PEPG: IL-1Ra | 365 | 0.146 | < 0.60 |
| R848: MIP-1α | 334 | 0.134 | < 0.60 |
| *S. aureus*: TNF-α | 333 | 0.134 | < 0.60 |
| *E. coli*: IL-6 | 332 | 0.133 | < 0.60 |
| *S. aureus*: MIP-1β | 309 | 0.124 | < 0.60 |
| *H. influenzae*: IL-10 | 307 | 0.123 | < 0.60 |
| *E. coli*: TNF-α | 305 | 0.122 | < 0.60 |
| PEPG: IL-8 | 267 | 0.107 | < 0.60 |
| *E. coli*: IL-10 | 206 | 0.083 | < 0.60 |

*Frequency is the proportion of successful ENR fits (n = 2,491) in which each variable was retained. Variables in bold meet the pre-specified stability threshold of 0.75.

**Supplementary Table 6. Elastic-net stability selection frequencies, FA — cytokines + full clinical.**

| Variable | Times selected | Selection frequency | Stability band |
| --- | --- | --- | --- |
| **Baby: Topical steroid treatment by 6m** | **2,453** | **0.985** | **≥ 0.90** |
| **Baby: Eczema by 6m** | **2,185** | **0.877** | **0.80–0.89** |
| **Baby: Daycare attendance** | **2,150** | **0.863** | **0.80–0.89** |
| R848: IL-1Ra | 1,644 | 0.660 | 0.60–0.69 |
| *S. pyogenes*: IP-10 | 1,554 | 0.624 | 0.60–0.69 |
| Pam3CSK4: IL-1Ra | 1,511 | 0.607 | 0.60–0.69 |
| Household: Parent(s) ANY atopic disease | 1,347 | 0.541 | < 0.60 |
| *H. influenzae*: IL-1Ra | 1,304 | 0.523 | < 0.60 |
| *E. coli*: MCP-1 | 1,223 | 0.491 | < 0.60 |
| Baby: Gestational age | 1,210 | 0.486 | < 0.60 |
| R848: IP-10 | 1,108 | 0.445 | < 0.60 |
| *E. coli*: MIF | 1,057 | 0.424 | < 0.60 |
| PEPG: IFN-γ | 1,043 | 0.419 | < 0.60 |
| *C. albicans*: IFN-γ | 990 | 0.397 | < 0.60 |
| LPS: IL-1β | 961 | 0.386 | < 0.60 |
| Baby: Male | 909 | 0.365 | < 0.60 |
| *S. aureus*: IFN-γ | 876 | 0.352 | < 0.60 |
| Household: 1 sibling | 842 | 0.338 | < 0.60 |
| Mother: Antibiotics last trimester pregnancy | 795 | 0.319 | < 0.60 |
| *H. influenzae*: IFN-γ | 791 | 0.318 | < 0.60 |
| *S. pyogenes*: IL-1Ra | 789 | 0.317 | < 0.60 |
| *S. pneumoniae*: MIP-1α | 777 | 0.312 | < 0.60 |
| *S. pyogenes*: IL-10 | 771 | 0.309 | < 0.60 |
| *E. coli*: IL-1Ra | 765 | 0.307 | < 0.60 |
| *E. coli*: MIP-1β | 753 | 0.302 | < 0.60 |
| LPS: IL-1Ra | 752 | 0.302 | < 0.60 |
| *H. influenzae*: MIP-1β | 747 | 0.300 | < 0.60 |
| Mother: University level education | 744 | 0.299 | < 0.60 |
| Household: ≥5 inhabitants | 738 | 0.296 | < 0.60 |
| *S. aureus*: IL-1β | 734 | 0.295 | < 0.60 |
| *E. coli*: IP-10 | 706 | 0.283 | < 0.60 |
| Baby: Caesarean section | 690 | 0.277 | < 0.60 |
| Baby: Vaginal delivery | 690 | 0.277 | < 0.60 |
| Baby: BCG | 686 | 0.275 | < 0.60 |
| *S. pneumoniae*: IP-10 | 677 | 0.272 | < 0.60 |
| Baby: Birth weight | 661 | 0.265 | < 0.60 |
| *S. pyogenes*: IL-1β | 660 | 0.265 | < 0.60 |
| *S. pneumoniae*: IL-8 | 657 | 0.264 | < 0.60 |
| r_ba_bf_at_cat>3mth -12m+ | 657 | 0.264 | < 0.60 |
| LPS: IL-10 | 654 | 0.263 | < 0.60 |
| *H. influenzae*: IP-10 | 652 | 0.262 | < 0.60 |
| Pam3CSK4: IFN-γ | 643 | 0.258 | < 0.60 |
| LPS: MCP-1 | 638 | 0.256 | < 0.60 |
| *L. monocytogenes*: MCP-1 | 615 | 0.247 | < 0.60 |
| *L. monocytogenes*: IL-1β | 613 | 0.246 | < 0.60 |
| *S. pneumoniae*: MCP-1 | 605 | 0.243 | < 0.60 |
| Mother: Age at participant's birth | 602 | 0.242 | < 0.60 |
| Baby: Birth season | 601 | 0.241 | < 0.60 |
| LPS: IP-10 | 601 | 0.241 | < 0.60 |
| *L. monocytogenes*: IL-10 | 587 | 0.236 | < 0.60 |
| Pam3CSK4: MIP-1α | 586 | 0.235 | < 0.60 |
| LPS: MIP-1α | 582 | 0.234 | < 0.60 |
| Household: Cat/Dog/Livestock | 574 | 0.230 | < 0.60 |
| *S. pneumoniae*: MIP-1β | 572 | 0.230 | < 0.60 |
| *S. pyogenes*: MCP-1 | 558 | 0.224 | < 0.60 |
| *S. pyogenes*: IL-6 | 557 | 0.224 | < 0.60 |
| R848: IL-1β | 537 | 0.216 | < 0.60 |
| *C. albicans*: IL-1β | 531 | 0.213 | < 0.60 |
| Mother: Antibiotics during labour | 522 | 0.210 | < 0.60 |
| *S. pyogenes*: TNF-α | 521 | 0.209 | < 0.60 |
| *S. pneumoniae*: IL-1β | 519 | 0.208 | < 0.60 |
| Baby: Vitamin D in hospital | 518 | 0.208 | < 0.60 |
| *S. aureus*: MCP-1 | 507 | 0.203 | < 0.60 |
| *S. pyogenes*: MIP-1α | 498 | 0.200 | < 0.60 |
| Pam3CSK4: IP-10 | 487 | 0.196 | < 0.60 |
| *S. aureus*: IP-10 | 487 | 0.196 | < 0.60 |
| Household: Smokers lived in house during pregnancy | 485 | 0.195 | < 0.60 |
| *E. coli*: IL-1β | 466 | 0.187 | < 0.60 |
| *S. pneumoniae*: IFN-γ | 465 | 0.187 | < 0.60 |
| Pam3CSK4: IL-1β | 461 | 0.185 | < 0.60 |
| *S. aureus*: IL-6 | 461 | 0.185 | < 0.60 |
| *H. influenzae*: MIP-1α | 453 | 0.182 | < 0.60 |
| Mother: Vitamin D during pregnancy | 449 | 0.180 | < 0.60 |
| *S. aureus*: MIP-1α | 445 | 0.179 | < 0.60 |
| *L. monocytogenes*: IL-1Ra | 439 | 0.176 | < 0.60 |
| Mother: Probiotics during pregnancy | 437 | 0.175 | < 0.60 |
| Household: 1 other child 5-18y | 437 | 0.175 | < 0.60 |
| Mother: GBS positive | 427 | 0.171 | < 0.60 |
| *C. albicans*: TNF-α | 423 | 0.170 | < 0.60 |
| *E. coli*: IFN-γ | 423 | 0.170 | < 0.60 |
| *S. pneumoniae*: IL-1Ra | 419 | 0.168 | < 0.60 |
| *S. pneumoniae*: IL-6 | 418 | 0.168 | < 0.60 |
| Baby: Birth order | 411 | 0.165 | < 0.60 |
| PEPG: IL-6 | 410 | 0.165 | < 0.60 |
| *L. monocytogenes*: IFN-γ | 409 | 0.164 | < 0.60 |
| *C. albicans*: IL-10 | 407 | 0.163 | < 0.60 |
| r_env_sibling_cat_pr2 or more | 406 | 0.163 | < 0.60 |
| *C. albicans*: IP-10 | 404 | 0.162 | < 0.60 |
| *S. pneumoniae*: TNF-α | 399 | 0.160 | < 0.60 |
| LPS: IL-8 | 398 | 0.160 | < 0.60 |
| *S. aureus*: IL-8 | 397 | 0.159 | < 0.60 |
| *S. pyogenes*: IL-8 | 382 | 0.153 | < 0.60 |
| *S. pneumoniae*: IL-10 | 382 | 0.153 | < 0.60 |
| PEPG: IL-1β | 375 | 0.150 | < 0.60 |
| *L. monocytogenes*: TNF-α | 374 | 0.150 | < 0.60 |
| *S. pyogenes*: MIP-1β | 372 | 0.149 | < 0.60 |
| Household: ≥2 other children 5-18y | 372 | 0.149 | < 0.60 |
| *S. aureus*: IL-10 | 365 | 0.146 | < 0.60 |
| *L. monocytogenes*: IL-8 | 363 | 0.146 | < 0.60 |
| Household: 3 or 4 inhabitants | 363 | 0.146 | < 0.60 |
| *S. pyogenes*: IFN-γ | 361 | 0.145 | < 0.60 |
| PEPG: IP-10 | 361 | 0.145 | < 0.60 |
| *C. albicans*: MCP-1 | 356 | 0.143 | < 0.60 |
| *L. monocytogenes*: IP-10 | 356 | 0.143 | < 0.60 |
| R848: IL-10 | 352 | 0.141 | < 0.60 |
| *L. monocytogenes*: IL-6 | 351 | 0.141 | < 0.60 |
| PEPG: MIP-1β | 340 | 0.137 | < 0.60 |
| *H. influenzae*: MCP-1 | 339 | 0.136 | < 0.60 |
| R848: MCP-1 | 338 | 0.136 | < 0.60 |
| R848: MIP-1β | 337 | 0.135 | < 0.60 |
| PEPG: MIG | 323 | 0.130 | < 0.60 |
| *L. monocytogenes*: MIP-1β | 302 | 0.121 | < 0.60 |
| *E. coli*: MIG | 293 | 0.118 | < 0.60 |
| *L. monocytogenes*: MIP-1α | 293 | 0.118 | < 0.60 |
| LPS: TNF-α | 283 | 0.114 | < 0.60 |
| LPS: MIP-1β | 280 | 0.112 | < 0.60 |
| LPS: IFN-γ | 274 | 0.110 | < 0.60 |
| Pam3CSK4: MIP-1β | 273 | 0.110 | < 0.60 |
| Pam3CSK4: TNF-α | 272 | 0.109 | < 0.60 |
| PEPG: MIP-1α | 270 | 0.108 | < 0.60 |
| *S. aureus*: TNF-α | 267 | 0.107 | < 0.60 |
| PEPG: TNF-α | 260 | 0.104 | < 0.60 |
| Pam3CSK4: IL-10 | 247 | 0.099 | < 0.60 |
| PEPG: MIF | 245 | 0.098 | < 0.60 |
| *E. coli*: MIP-1α | 243 | 0.098 | < 0.60 |
| Pam3CSK4: IL-6 | 239 | 0.096 | < 0.60 |
| PEPG: MCP-1 | 239 | 0.096 | < 0.60 |
| *C. albicans*: IL-1Ra | 226 | 0.091 | < 0.60 |
| R848: TNF-α | 223 | 0.089 | < 0.60 |
| *C. albicans*: IL-6 | 219 | 0.088 | < 0.60 |
| *H. influenzae*: IL-1β | 213 | 0.086 | < 0.60 |
| *E. coli*: IL-8 | 211 | 0.085 | < 0.60 |
| *C. albicans*: MIP-1β | 208 | 0.084 | < 0.60 |
| Pam3CSK4: MCP-1 | 208 | 0.084 | < 0.60 |
| *S. aureus*: MIP-1β | 204 | 0.082 | < 0.60 |
| R848: IL-6 | 188 | 0.075 | < 0.60 |
| *C. albicans*: IL-8 | 187 | 0.075 | < 0.60 |
| PEPG: IL-1Ra | 174 | 0.070 | < 0.60 |
| R848: MIP-1α | 174 | 0.070 | < 0.60 |
| *C. albicans*: MIP-1α | 168 | 0.067 | < 0.60 |
| *H. influenzae*: IL-8 | 167 | 0.067 | < 0.60 |
| *H. influenzae*: IL-6 | 165 | 0.066 | < 0.60 |
| PEPG: IL-8 | 152 | 0.061 | < 0.60 |
| Pam3CSK4: IL-8 | 147 | 0.059 | < 0.60 |
| PEPG: IL-10 | 147 | 0.059 | < 0.60 |
| *S. aureus*: IL-1Ra | 147 | 0.059 | < 0.60 |
| R848: IFN-γ | 140 | 0.056 | < 0.60 |
| R848: IL-8 | 116 | 0.047 | < 0.60 |
| *H. influenzae*: IL-10 | 114 | 0.046 | < 0.60 |
| *E. coli*: TNF-α | 107 | 0.043 | < 0.60 |
| LPS: IL-6 | 86 | 0.034 | < 0.60 |
| *H. influenzae*: TNF-α | 81 | 0.032 | < 0.60 |
| *E. coli*: IL-10 | 75 | 0.030 | < 0.60 |
| *E. coli*: IL-6 | 51 | 0.020 | < 0.60 |

*Frequency is the proportion of successful ENR fits (n = 2,491) in which each variable was retained. Variables in bold meet the pre-specified stability threshold of 0.75.

1.	Williams HC, Burney PG, Hay RJ, et al. The U.K. Working Party's Diagnostic Criteria for Atopic Dermatitis. I. Derivation of a minimum set of discriminators for atopic dermatitis. *Br J Dermatol* 1994; **131**(3): 383-96.

2.	Williams HC, Burney PG, Pembroke AC, Hay RJ. The U.K. Working Party's Diagnostic Criteria for Atopic Dermatitis. III. Independent hospital validation. *Br J Dermatol* 1994; **131**(3): 406-16.

3.	Charman CR, Venn AJ, Williams HC. The patient-oriented eczema measure: development and initial validation of a new tool for measuring atopic eczema severity from the patients' perspective. *Arch Dermatol* 2004; **140**(12): 1513-9.

4.	Messina NL, Gardiner K, Pittet LF, et al. Neonatal BCG Vaccination for Prevention of Allergy in Infants: The MIS BAIR Randomised Controlled Trial. *Clin Exp Allergy* 2024.

5.	Freyne B, Donath S, Germano S, et al. Neonatal BCG Vaccination Influences Cytokine Responses to Toll-like Receptor Ligands and Heterologous Antigens. *J Infect Dis* 2018; **217**(11): 1798-808.

6.	Freyne B, Messina NL, Donath S, et al. Neonatal BCG Vaccination Reduces Interferon-gamma Responsiveness to Heterologous Pathogens in Infants From a Randomized Controlled Trial. *J Infect Dis* 2020; **221**(12): 1999-2009.
